# PredictSNP2: A Unified Platform for Accurately Evaluating SNP Effects by Exploiting the Different Characteristics of Variants in Distinct Genomic Regions

**DOI:** 10.1371/journal.pcbi.1004962

**Published:** 2016-05-25

**Authors:** Jaroslav Bendl, Miloš Musil, Jan Štourač, Jaroslav Zendulka, Jiří Damborský, Jan Brezovský

**Affiliations:** 1 Loschmidt Laboratories, Department of Experimental Biology and Research Centre for Toxic Compounds in the Environment RECETOX, Masaryk University, Brno, Czech Republic; 2 Department of Information Systems, Faculty of Information Technology, Brno University of Technology, Brno, Czech Republic; 3 International Clinical Research Center, St. Anne’s University Hospital Brno, Brno, Czech Republic; University of Canterbury, NEW ZEALAND

## Abstract

An important message taken from human genome sequencing projects is that the human population exhibits approximately 99.9% genetic similarity. Variations in the remaining parts of the genome determine our identity, trace our history and reveal our heritage. The precise delineation of phenotypically causal variants plays a key role in providing accurate personalized diagnosis, prognosis, and treatment of inherited diseases. Several computational methods for achieving such delineation have been reported recently. However, their ability to pinpoint potentially deleterious variants is limited by the fact that their mechanisms of prediction do not account for the existence of different categories of variants. Consequently, their output is biased towards the variant categories that are most strongly represented in the variant databases. Moreover, most such methods provide numeric scores but not binary predictions of the deleteriousness of variants or confidence scores that would be more easily understood by users. We have constructed three datasets covering different types of disease-related variants, which were divided across five categories: (i) regulatory, (ii) splicing, (iii) missense, (iv) synonymous, and (v) nonsense variants. These datasets were used to develop category-optimal decision thresholds and to evaluate six tools for variant prioritization: CADD, DANN, FATHMM, FitCons, FunSeq2 and GWAVA. This evaluation revealed some important advantages of the category-based approach. The results obtained with the five best-performing tools were then combined into a consensus score. Additional comparative analyses showed that in the case of missense variations, protein-based predictors perform better than DNA sequence-based predictors. A user-friendly web interface was developed that provides easy access to the five tools’ predictions, and their consensus scores, in a user-understandable format tailored to the specific features of different categories of variations. To enable comprehensive evaluation of variants, the predictions are complemented with annotations from eight databases. The web server is freely available to the community at http://loschmidt.chemi.muni.cz/predictsnp2.

This is a *PLOS Computational Biology* Software paper.

## Introduction

The rapid development and falling costs of sequencing technologies have enabled the study of human genetic variants on a large scale [1]. Genome sequencing projects have generated a very large catalog of human genetic variations, but the interpretation of these data remains challenging. In particular, it is difficult to determine the functional impact of variants on individuals [2–4] and sub-populations [5,6]. These difficulties have become more pronounced and important as the scope of analysis has expanded from Mendelian disorders [7,8] to complex diseases such as diabetes [9]. Improvements in sequencing technologies have also allowed researchers to move beyond studying associations in the exome: over the last decade, several large-scale genome projects have provided evidence that the concept of “junk DNA” is flawed and at least 80% of the human genome is functional [10]. The Encyclopedia of DNA Elements (ENCODE) [10] and Epigenomics Roadmap [11] projects have released comprehensive maps of regulatory elements such as transcription factor binding sites, chromatin regulators, and regions of histone modification. These annotations are available for many different cells and tissue types, and provide an opportunity to detect new pathogenic variants. The disease mechanisms associated with some of these variants can be linked to perturbations in specific regulatory elements that alter gene expression [12,13]. Although only a few Mendelian phenotypes have been mapped exclusively to genetic variants outside the exome [14], it is likely that many remain to be discovered. At present, about 50% of all 3,152 known Mendelian phenotypes have no known association with coding regions [8] and thus represent promising candidates for further investigation. Furthermore, genome-wide association studies (GWAS) have identified over twenty thousand variants, of which over 90% occurred in non-coding regions [15]. These variants have been associated with common diseases in which lifestyle and environmental factors play important roles [16]. This finding supports the hypothesis that most trait-associated variants with weak effects are non-coding [1].

Computational analysis is very important for prioritizing variants. While there are many tools dedicated to predicting the effects of missense variations [1,17,18], only a handful have been developed for analysis of non-coding variants. Because strong descriptors were not widely available in the past, the first nucleotide-based tools relied exclusively on evolutionary conservation in their analyses [19–21]. Unfortunately, the predictive performance of these tools is limited by the high evolutionary turnover of regulatory elements [22,23], which makes it harder to derive a significant signal from their degree of conservation than is the case for coding regions. The release of data from genome projects subsequently enabled the development of a new generation of tools [24–32]. While all of the second-generation tools take advantage of new functional annotations of the genome and offer superior performance to conservation-based tools, their ability to provide accurate and interpretable estimates of deleteriousness for all genome variations is often limited by two factors. First, they do not account for the existence of different types of variations during the learning phase, so their results are biased towards missense variants, which are over-represented in the variation databases. Second, most of them do not provide clear statements about the deleteriousness of analyzed variants or human-readable confidence scores. Instead, they report decimal values from numeric ranges without fixed decision thresholds, making interpretation of their results difficult.

Here we report the construction of three balanced datasets covering different types of disease-related variants. To study the performance of individual tools in more detail, each dataset was further divided into five categories: (i) regulatory, (ii) splicing, (iii) missense, (iv) synonymous, and (v) nonsense variants. The datasets representing these categories were evaluated using six prediction tools and used to develop category-optimal decision thresholds. The use of these optimized thresholds with the predictive tools often significantly increases their performance relative to that achieved with a general single-threshold approach. In addition, we have developed a web interface providing easy access to binary predictions and uniform confidence values for the five best-performing prediction tools and their consensus. These predictions are supplemented with information gathered from eight publically available databases. Herein introduced tool, PredictSNP2, represents a natural extension of previously published PredictSNP1 tool [33]. PredictSNP1 offers its users a consensus score based on the output of six different amino acid-based predictors. Because of the nature of the tools whose results are combined to generate its consensus, PredictSNP1 can only be used to analyze substitutions in an amino acid sequence. PredictSNP2 complements PredictSNP1 by evaluating the effects of nucleotide variants located in any region of the genome.

## Design and Implementation

### Datasets and data preprocessing

**Fig 1:**
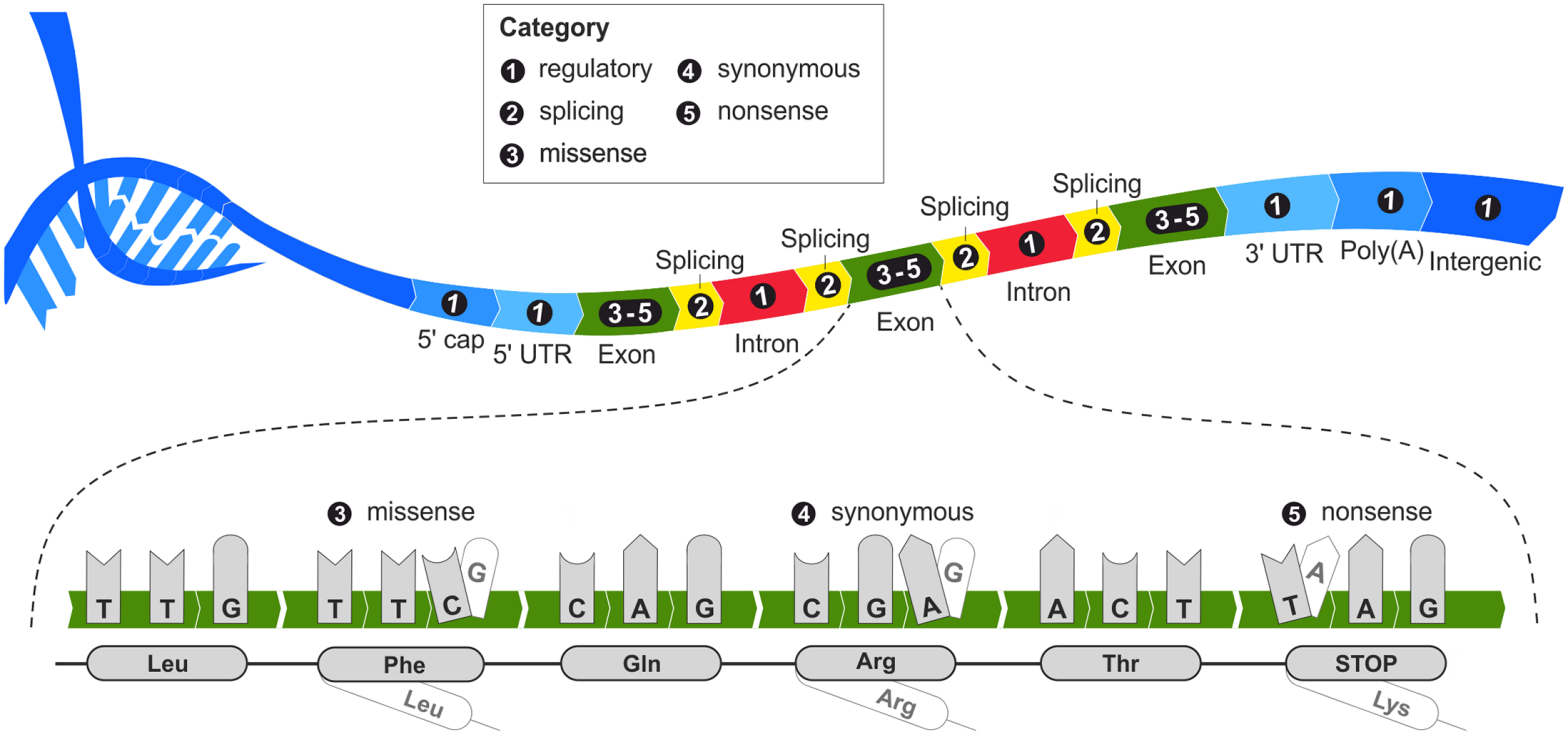
Categorization of variants based on their location within the genome and their type.

A collection of three datasets covering different types of pathogenic variants associated with Mendelian, complex, and cancer diseases was constructed. This division was chosen to reflect the different genetic basis of these diseases [9] and the differences in the extent of their phenotypic effects [34]. A dataset of variants associated with Mendelian diseases was created using all variants annotated as pathogenic or likely pathogenic in NCBI ClinVar [35], a manually curated database of genotype-phenotype relationships. Information on variants associated with complex human diseases (*p*-value < 10^−8^) was obtained from the NHGRI GWAS catalog [15], a collection of all publicly available genome-wide association studies. To compile the dataset of somatic cancer variants, we extracted all records with confirmed somatic status present in at least two different samples from the COSMIC database [36]. Each disease-related dataset was then split into five subsets by classifying the variants according to their functional consequences and location within the genome as determined by ANNOVAR [37] (Fig 1). The decision to use fine-grained variant categorization was motivated by the observation that the classification features used by the evaluated tools exhibit different signals within different categories [38]. Finally, these categorized pathogenic variants were supplemented with their neutral counterparts from the VariSNP database [39]. In addition to the standard VariSNP procedure of removing all overlaps with disease-related records from ClinVar [35], Swiss-Prot [40] and PhenCode [41], we also filtered out all variants present in the COSMIC [36] and NHGRI GWAS catalogs [15]. We used the distance-based approach introduced by Ritchie *et al*. to construct the neutral subsets [25], selecting the closest available neutral variant in the neighborhood of each individual deleterious variant. This approach can be expected to yield balanced datasets if one assumes that the neutral variants should reliably sample the overall background. Because the advantage of using category-specific thresholds or consensus scores should not be evaluated against the same datasets used for such optimizations [42], we split all of the individual category datasets into training and testing subsets based on the entries’ dates of submission. To ensure that the testing dataset excluded information that may have previously been used to train individual tools, it contained only variations submitted after December 2014. While the variants in the non-exonic categories were divided randomly across these subsets, the corresponding protein sequences representing exonic regions were clustered by CD-HIT [43] at the level of 50% sequence identity to ensure that variants occurring in similar proteins were assigned to the same set. The final versions of the datasets are available in the supporting information (S1–S3 Datasets).

### Prediction tools and databases

Six prediction tools were selected for evaluation, optimization and eventual integration into the PredictSNP2 web portal (see S1 Table). These tools had to satisfy the following criteria: (i) to be capable of predicting the effects of a nucleotide substitution anywhere within the human genome, (ii) to be available as a stand-alone application or to provide pre-calculated scores for all possible substitutions, (iii) to have a higher level of complexity than established first-principles approaches. The latter criterion prevented the inclusion of tools that base their predictions solely on evolutionary data. This was done because the rapid evolution and varied evolutionary patterns observed outside the protein-coding regions of the genome [21] mean that evolutionary constraints do not provide sufficient discriminatory power by themselves for non-coding regions, although they can be useful when combined with other features. All six selected tools benefit from the availability of functional annotations from the ENCODE project [10]. They represent diverse predictive approaches leveraging different training datasets, machine learning models, and combinations of decision features. CADD [24] estimates the deleteriousness of variants, a property correlated with both molecular functionality and pathogenicity. Its predictions are based on a logistic regression model that takes into account evolutionary conservation, regulatory and transcript information, and protein-level scores. The CADD classifier was trained on a newly constructed dataset of mutations including a subset of approximately 15 million putatively neutral variants derived from observed differences between the human and chimpanzee genomes, and a second subset of approximately 15 million simulated variants that was enriched in deleterious variants because it had not been subject to natural selection. A similar approach was used with DANN [26], a deep neural network-based classifier with the ability to capture non-linear relationships among features. FATHMM-MKL [27] assesses the functional impact of variants using an SVM model, which was trained on a set of literature-derived pathogenic variants drawn from the Human Gene Mutation Database (HGMD) [44] and neutral common variants drawn from the 1000 Genome Project [45]. Data from the same sources was used to build a training dataset for the GWAVA [25] tool, which is based on a random forest classifier and is designed for the analysis of regulatory variants. FunSeq2 [32] uses an empirical scoring system that integrates evolutionary constraints, epigenetic data and knowledge of transcription-binding motifs to assess the impact of variants. The weights of selected features were derived from mutation patterns observed in the 1000 Genomes polymorphism data. Finally, FitCons [28] defines clusters of similar functional genomic signals, which are termed fingerprints, and then estimates the functional impact of variants with the same fingerprint on the basis of allele frequency distributions in human populations. To help users navigate the wide range of available online data sources, the analyzed variants are supplemented with links to the corresponding entries in eight separate databases (S2 Table): dbSNP [46], which provides general information about individual variants; ClinVar [35] and Online Mendelian Inheritance in Man (OMIM) [47], which provide interpretations of the variants’ relationships with human health; HaploReg [48] and RegulomeDB [49], which provide access to a variety of ENCODE annotations [10]; NCBI GenBank [50], which provides the sequence corresponding to the variant; and the UCSC Genome browser [51] or Ensembl Genome browser [52], which display the sequence together with information from various biological databases.

### Performance evaluation

The performance of the six nucleotide-based tools and the consensus predictions generated with PredictSNP2 was evaluated using standard statistical metrics, as summarized in the supporting information (S1 Text). Because only FATHMM and GWAVA provide binary predictions, we derived optimal decision thresholds for all pairs of tools and categories of variants that can be used to obtain binary predictions from the output of CADD, DANN, FitCons and FunSeq2. These thresholds were set to provide the highest normalized accuracy with the training subsets for any given category. We also compared the performance of selected nucleotide-based prediction tools to that of some protein-level tools, which were selected on the basis of our previous study [33] that focused on identifying disease-related amino acid mutations. The chosen protein-level tools were MAPP [53], PhD-SNP [54], PolyPhen-1 [55], PolyPhen-2 [56], SIFT [57], SNAP [58], and meta-tool PredictSNP1 [33]. To enable this comparison, ANNOVAR was used to convert original nucleotide variants in non-synonymous exonic categories present in our datasets into amino acid format, and to retrieve identifiers of the amino acid sequences of the corresponding gene products. These sequences were retrieved using NCBI eUtils (http://eutils.ncbi.nlm.nih.gov), and represent a necessary input for protein-based tools. To avoid potential bias in favor of the protein-based tools, all amino acid mutations at positions overlapping with the training datasets of the protein-based tools were discarded. The final dataset used in this comparative analysis is provided in the supporting information (S4 and S5 Datasets).

### Consensus classifier

The five best-performing tools were integrated into the consensus classifier PredictSNP2 using the method developed previously [33]. Briefly, the consensus was determined on the basis of a majority vote, with the individual tools’ votes being weighted by their confidences. In the present study, the uniform confidences were derived separately for each tool and category of variants using a relationship between the tool’s raw score and its accuracy when tested against a training subset representing the category of interest. All of the evaluated mutations from the training subset were sorted by their raw score and partitioned into 66 bins of equal size. These bins were subsequently averaged over eleven neighboring bins. Two separate transformation functions were developed for deleterious and neutral predictions to account for differences in the relationships between the confidence score and the observed accuracy for these two prediction classes. The category-specific decision thresholds for the individual integrated tools were used to distinguish between the neutral and deleterious cases. In this way, the scores of integrated tools were normalized onto a single scale, facilitating comparisons. After the overall predictions and corresponding transformed confidence scores had been obtained, the PredictSNP2 consensus score was calculated. Finally, the corresponding binary prediction and uniform confidence score was obtained also for the PredictSNP2 consensus score in the same way as described for the individual integrated tools.

## Results

### Construction of the Mendelian disease dataset

**Fig 2:**
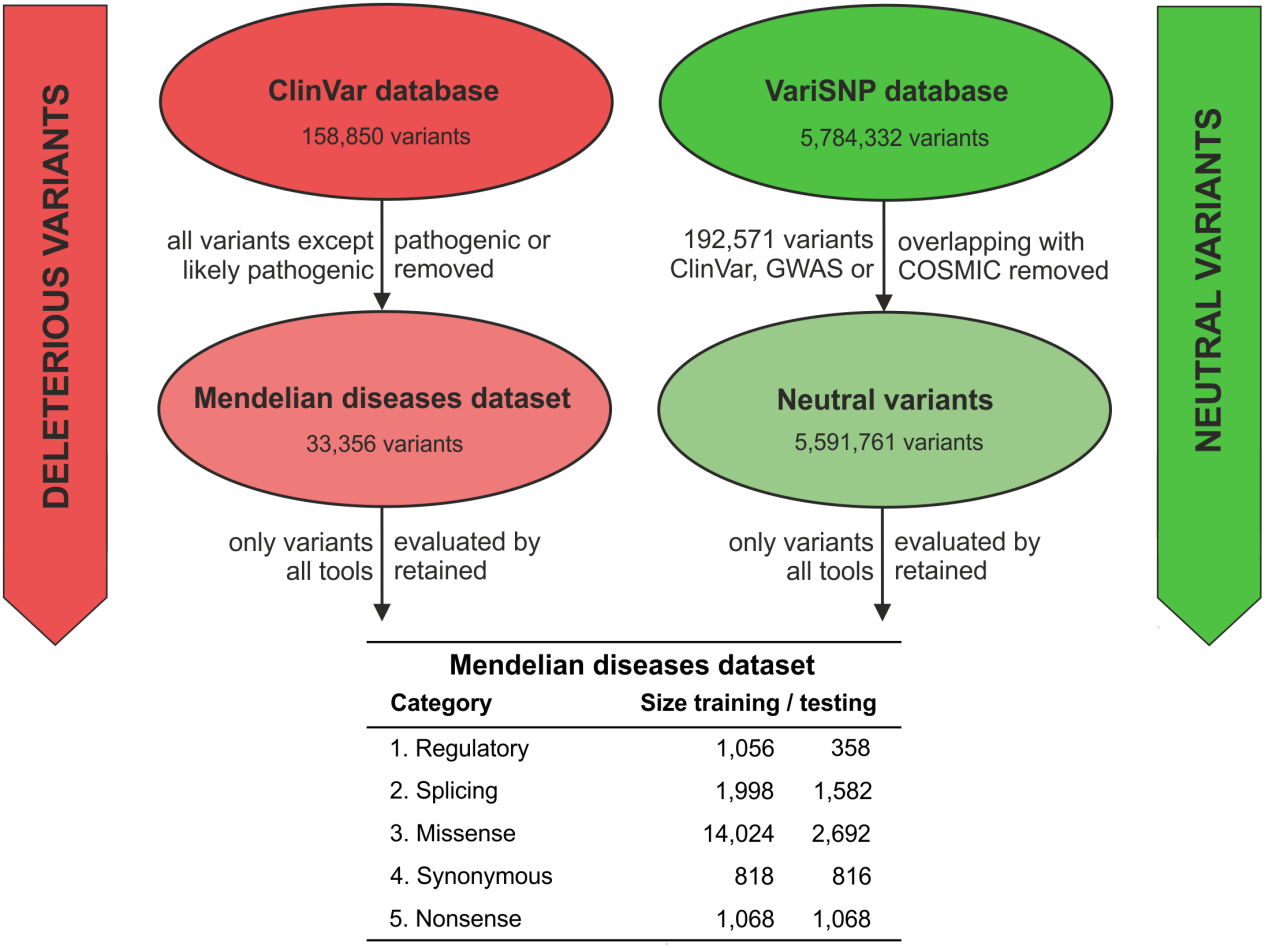
Workflow diagram describing the construction of the dataset of variants related to Mendelian diseases. The dataset was prepared by combining deleterious variants from the ClinVar database with neutral variants from the VariSNP database. The resulting dataset was then divided into independent training and testing subsets for each individual category of variants.

The dataset consisted of Mendelian disease-related variants and their neutral counterparts; in total, it included 25,480 variants. These variants were divided into separate categories according to their location and type, i.e. into regulatory, splicing, missense, synonymous and nonsense variants (Fig 2). This step is justified by the large differences in the numbers of variants representing each category, which ranged from the low hundreds to over ten thousand, as well as by the different characteristics of individual categories [38]. Each category was then subdivided into training and testing subsets. The training subsets were used to compute category-optimal thresholds for individual tools and to derive the procedure for computing the consensus score, while the test subset was used to independently evaluate their performance. For the missense and synonymous variant categories, an additional criterion of at most 50% protein sequence identity was imposed to ensure that all variants representing highly similar protein sequences were placed in the same subset.

### Development of category-optimal thresholds

**Fig 3:**
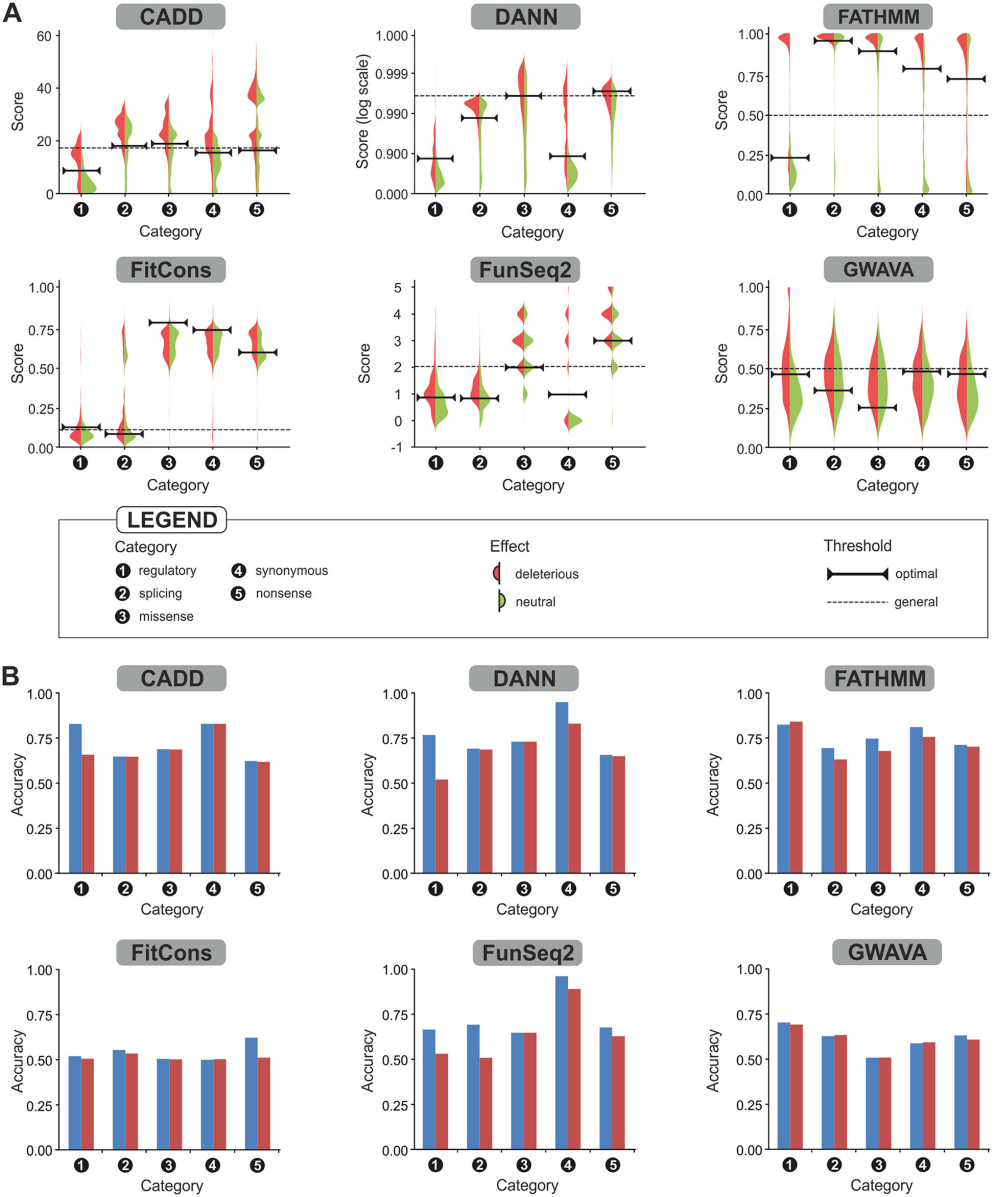
The use of category-optimal thresholds improves the predictive performance of individual tools by increasing their ability to capture differences in the distribution of prediction scores for the different categories of variants. (A) Distribution of scores for deleterious and neutral variants provided by each evaluated tool for individual categories of variants from the training subsets of the Mendelian diseases dataset. The locations of the general and category-optimal thresholds used to obtain predictions are shown for each tool. (B) Normalized accuracies achieved by individual tools when using category-optimal (blue bars) and general (red bars) thresholds, evaluated using testing subsets of the Mendelian diseases dataset.

All variants present in the constructed datasets were evaluated using the six investigated tools separately. There were important differences between the raw score distributions obtained with the individual tools for different categories of variants in the Mendelian diseases dataset (Fig 3A). That is to say, the score distribution achieved for a given variant category with a particular tool differed substantially from the distributions assigned to other categories by the same tool. More importantly, these category-specific distributions were frequently observed for both deleterious and neutral variants, suggesting a need for category-specific thresholds to achieve optimal separation of deleterious and neutral variants. Category-optimal thresholds were derived from the training subsets of all categories for all six individual tools to adjust the binary predictions with respect to observed differences in the raw scores between the individual categories. The positive effect of category-specific optimization was detected for at least half of the tool-category pairs (Fig 3B and S3 Table). The most prominent effects were observed for the categories that exhibited the most dissimilar score distributions for a given tool (Fig 3A). The greatest increase in the average accuracy resulting from the use of category-optimal thresholds was observed in the case of regulatory variants, for which accuracy increased by 9%. Smaller increases between 1% and 4% were observed for all remaining variant categories (Fig 3B and S3 Table). The tools whose predictive power was most strongly increased by the use of category-specific thresholds were FunSeq2 and DANN, whose average accuracies rose by 9% and 8%, respectively (S3 Table). Conversely, the threshold optimization generally had negligible effects on the performance of GWAVA. The greatest increases in accuracy were observed for regulatory variants in the case of DANN (25%), CADD (17%) and FunSeq2 (13%), and for splicing variants in the case of FunSeq2 (18%; see Fig 3B).

### Performance of individual nucleotide-based prediction tools with category-optimal thresholds

A comprehensive evaluation of the integrated tools revealed that most were well capable of differentiating between Mendelian disease-related variants with neutral and deleterious effects (Table 1 and S1 Fig). However, GWAVA and FitCons exhibited significantly lower accuracies and areas under the receiver operating characteristic curve (AUC) than the other tools. The overall accuracies of these two tools across all individual categories were 58% and 53%, respectively. The performance of FitCons was considered insufficient to warrant its further use in the remainder of the study. The very low performance of GWAVA for the missense and synonymous mutation categories can be partially explained by its focus on the evaluation of regulatory variants, the only category for which it achieved a good accuracy (70%). The four remaining tools exhibited very satisfactory overall accuracies between 69% and 74%. Across the five best-performing tools, variants from the synonymous and regulatory categories were discriminated with appreciably higher average accuracies (82% and 75%, respectively) than variants of other types.

**Table 1:** Performance of individual prediction tools employing category-optimal thresholds and their PredictSNP2 consensus score for individual variant categories, evaluated using the testing subset of variants associated with Mendelian diseases.

Performance metrics ^a^	Category	CADD	DANN	FATHMM	FitCons	FunSeq2	GWAVA	PredictSNP2 consensus ^b^
Accuracy	1. Regulatory	0.82	0.76	0.82	0.52	0.66	0.70	0.86
	2. Splicing	0.64	0.69	0.69	0.55	0.69	0.63	0.75
	3. Missense	0.68	0.73	0.74	0.50	0.64	0.51	0.77
	4. Synonymous	0.83	0.95	0.81	0.50	0.96	0.59	0.96
	5. Nonsense	0.62	0.65	0.71	0.62	0.67	0.63	0.72
	**Overall**	**0.69**	**0.73**	**0.74**	**0.53**	**0.70**	**0.58**	**0.79**
Area under the receiver operating characteristic curve ^c^	1. Regulatory	0.88	0.83	0.89	0.52	0.70	0.76	0.87
	2. Splicing	0.69	0.74	0.74	0.49	0.72	0.70	0.80
	3. Missense	0.77	0.76	0.79	0.53	0.66	0.51	0.80
	4. Synonymous	0.90	0.96	0.86	0.51	0.96	0.61	0.98
	5. Nonsense	0.65	0.69	0.75	0.65	0.72	0.70	0.78
	**Overall**	**0.73**	**0.74**	**0.76**	**0.51**	**0.68**	**0.61**	**0.83**

^a^ For a detailed evaluation, see S4 Table.

^b^ The performance of the optimal consensus for given category, for details see Table 2 and S7 Table.

^c^ Receiver operating characteristic curves are depicted in S1 Fig.

To investigate the diversity of predictions provided by the five best-performing tools, we compared them in a pairwise fashion. S5 Table shows the correlations of the raw scores within the individual variant categories. The highest correlations were observed for the CADD & DANN, CADD & FATHMM, and DANN & FATHMM pairs, reaching Spearman correlation coefficient over 0.6 across all categories on average. Such high correlation could be considered undesirable because we wanted to include a diverse set of tools whose predictions err on different subsets of variants [59]. However, the high correlations of those three couples were mainly due to their agreement on correctly predicted cases, which represented around 63% of the total on average (S6 Table). More importantly, we only rarely observed agreement between any pair of the five best-performing tools on an incorrect prediction (S6 Table). This observation coupled with the good overall performance of the five individual tools provided a sound basis for their integration into a consensus classifier.

### Development of PredictSNP2 consensus score

In our previous work on protein-based tools, we noted that classification based on a “majority vote” of individual tools, weighted by their uniform confidence values, offered consistently better performance than any integrated tool when tested against three independent and diverse datasets [33]. We therefore decided to utilize a similar confidence-weighted majority vote approach to develop a consensus scoring procedure for the five best-performing nucleotide-based tools (CADD, DANN, FATHMM, FunSeq2 and GWAVA). Since the predictive performance of the individual tools varied significantly over the different categories, we first tested the value of adding more tools into the consensus for each category (Table 2). Most of the developed consensus scores, which were constructed by combining two to five tools, performed better than the best individual tool for the evaluated category (Table 2). For individual categories, the best consensus was more accurate than the best integrated tool by 1% to 6%, with the exception of synonymous category where the consensus performed equally well as the best integrated tool. For splicing category, the best consensus exhibited higher accuracy (by 6%) and AUC (by 0.06) than the best integrated tool. It was not always beneficial to include all of the tools in the consensus, however. For regulatory, missense and synonymous categories, we even observed that including less accurate tools reduced the accuracy of the consensus. This was especially pronounced in the case of regulatory variants, for which the inclusion of GWAVA and FunSeq2 tools reduced the accuracy of the consensus by 2%. Such decrease could be expected due to the much low predictive power of both these tool for this category. In addition to the improvements in accuracy and AUC values, the benefit of combining predictions from individual tools into robust PredictSNP2 consensus scores is demonstrated by the fact that the individual tools that perform best for one variant category often perform only moderately well or even poorly for others, whereas the PredictSNP2 consensus consistently provides the most accurate predictions (Tables 1 and 2).

**Table 2:** Performance of different consensus scores for specific variant categories, evaluated using the testing subset of variants associated with Mendelian diseases.

Performance metrics ^a^	Category	PredictSNP2 consensus ^b^				The best individual tool ^d^	
		5 tools ^c^	4 tools ^c^	3 tools ^c^	2 tools ^c^		
Accuracy	1. Regulatory	0.84	0.85	**0.86**	0.85	0.82	CADD
	2. Splicing	**0.75**	0.74	0.75	0.70	0.69	FATHMM
	3. Missense	0.76	0.76	0.76	**0.77**	0.74	FATHMM
	4. Synonymous	0.95	**0.96**	0.96	0.95	0.96	FunSeq2
	5. Nonsense	**0.72**	0.71	0.71	0.70	0.71	FATHMM
Area under the receiver operating characteristic curve	1. Regulatory	0.88	0.89	**0.87**	0.87	0.89	FATHMM
	2. Splicing	**0.80**	0.80	0.80	0.72	0.74	FATHMM
	3. Missense	0.80	0.82	0.81	**0.80**	0.79	FATHMM
	4. Synonymous	0.97	**0.98**	0.98	0.97	0.96	DANN
	5. Nonsense	**0.78**	0.76	0.75	0.72	0.75	FATHMM

^a^ For a detailed evaluation, see S7 Table.

^b^ The best-performing consensus in each category is highlighted in bold.

^c^ Tools included in a particular consensus are listed in S7 Table.

^d^ The performance metric and name of the best-performing tool in a given category.

### Comparison of nucleotide-based and protein-based tools

**Fig 4:**
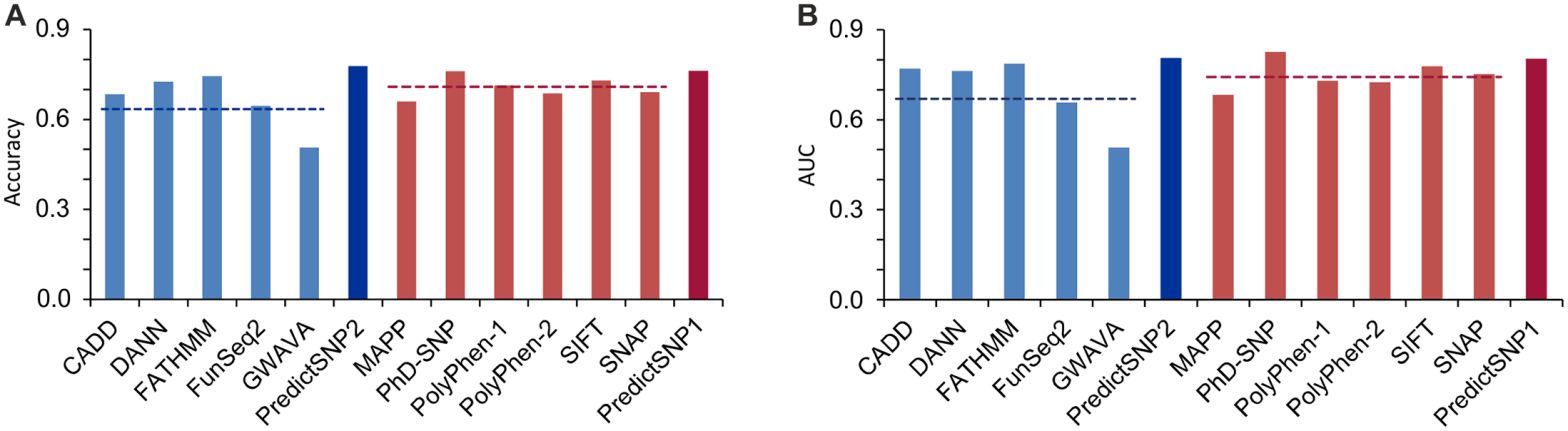
Performance of nucleotide-based and protein-based prediction tools and their consensuses, evaluated using the dataset of variants associated with Mendelian diseases. (A) Observed normalized accuracy and (B) area under the receiver operating characteristic curve (AUC) values are shown as blue and red bars for nucleotide- and protein-based tools and their consensuses, respectively. The horizontal dashed lines represent average performance values for each tool type.

The performance of five integrated nucleotide-based tools and their PredictSNP2 consensus scores was compared with that of six protein-based prediction tools and their PredictSNP1 consensus scores using the testing subset of missense variants. The accuracies of the protein-based tools (66–76%) were greater than those for the nucleotide-based predictors on average (51–74%; see Fig 4 and S8 Table). On the other hand, the performance of the best nucleotide-based tools, FATHMM and DANN, was comparable to the second and third best-ranked protein-based tools SIFT and PolyPhen-1, respectively. Moreover, the performances of the PredictSNP1 and PredictSNP2 consensus scores were similar for the evaluated missense variants (Fig 4 and S8 Table). Similar trends were observed in two recent comprehensive evaluations of various protein- and nucleotide-based predictors [18,60].

### Venturing beyond Mendelian variants

In addition to variants associated with Mendelian diseases, we wanted to assess the extent to which the integrated tools and their consensus scores can be utilized to evaluate variants implicated in complex diseases and somatic cancers because in these ailments the signal from genetic factors is often suppressed by the effects of external environmental factors [9,34]. To this end, we constructed two additional datasets containing variants associated with either complex diseases (12,050 variants) or somatic cancers (142,722 variants) by following the same protocol as for Mendelian diseases (S2 Fig). Although the disease-associated variants present in the three compiled datasets originated from different sources, there were partial overlaps among them (S3 Fig). The largest one was observed between the datasets of Mendelian and cancer diseases, which shared 140 deleterious variants. The presence of such overlaps is unsurprising because the clinical co-occurrence of certain Mendelian diseases and cancers can be tied to the same genetic variants [61,62]. In contrast to the situation with the Mendelian disease dataset, some of subsets representing the individual categories were assigned only a very low number of variants (S2 Fig), preventing any sensible performance evaluation for these categories

In the case of complex diseases, only the regulatory variants category included enough cases for analysis (S2 Fig). Interestingly, none of the five tested tools exhibited any discriminatory power whatsoever for this category (S9 Table), which stands in stark contrast to their very good performance for Mendelian variants in the same category (Table 1). Slightly better results were observed for somatic cancers, for which all categories bar that of splicing variants contained enough entries for evaluation (S2 Fig). For regulatory, missense and nonsense variants, the best tools achieved accuracies exceeding 60% as well as AUCs above 0.6 (S10 Table). However, such performance could still limit the tools’ applicability even for the purpose of variant prioritization. We also evaluated the performance of the protein-based tools with the missense variants from the cancer dataset (S8 Table and S4 Fig). In this case, neither protein-based tools nor their consensus score PredictSNP1 provided more reliable predictions than their nucleotide-based counterparts. The considerably lower predictive power of the investigated nucleotide-based tools on the complex disease and cancer datasets indicates that these tools and the PredictSNP2 consensus should only be applied to Mendelian diseases in order to ensure reliable predictions. More specialized tools and strategies focused on complex diseases [16] and cancers [63,64] should be used in other cases.

### Description of the web server

**Fig 5:**
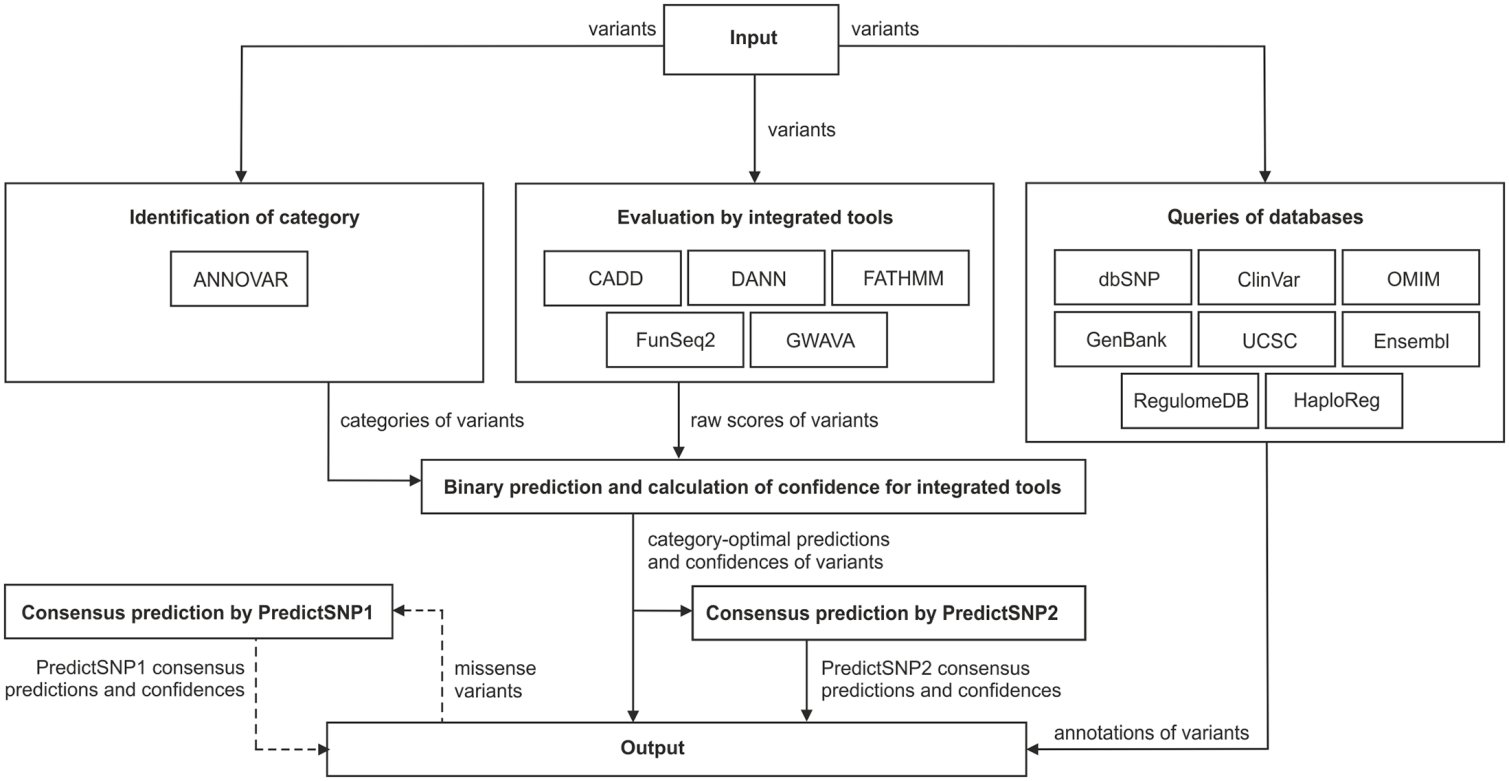
Workflow diagram of the PredictSNP2 webserver. Upon submission of input variants, evaluation is performed with the integrated prediction tools. The raw scores produced by individual tools are transformed into overall decisions about deleteriousness and interpretable confidence scores according to the category of variants detected by ANNOVAR. In addition, links to relevant databases and on-line tools are provided to allow the user to better understand the genomic context and potential function of the corresponding genome region. Optionally, evaluation of missense mutations by PredictSNP1 can be requested.

**Fig 6:**
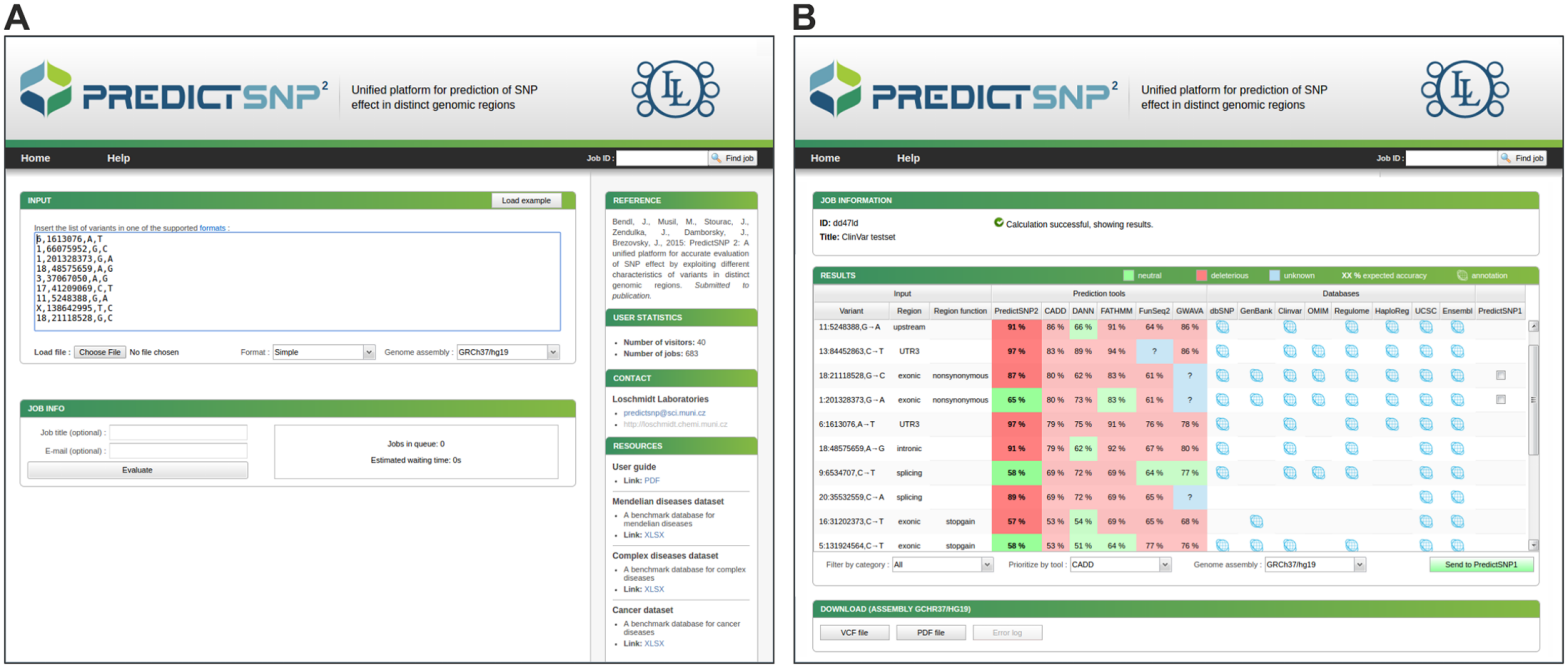
The graphical user interface of the PredictSNP2 webserver. (A) On the input page, variants to be analyzed can be provided in several established formats using one of two reference genome assemblies. (B) On the output page, the predictions of individual tools and their PredictSNP2 consensus score are reported together with links to the eight relevant databases.

Three of the five integrated prediction tools evaluated in this study are currently available as web servers. However, only CADD and FunSeq2 permit the uploading of files containing lists of variants to be analyzed and are thus suitable for large-scale queries. In contrast, FATHMM and GWAVA only permit variant querying via their web forms. DANN results are only available as pre-calculated files, which reduces the tool’s user-friendliness. To facilitate access to the predictions of all five integrated tools, we developed a web interface that enables the comfortable submission of large batches of variants. The interface also provides easily interpretable results for all individual tools together with the links to the relevant databases and on-line services (Fig 5). The variants to be analyzed can be input into a web form as a plain text or uploaded as a file. Variant data in multiple formats can be detected automatically, including the Variant Call Format (VCF) [65], Human Genome Variation Society (HGVS) format [66], and Genome Variation Format (GVF) [67]. Moreover, the user can switch between the two types of reference genome assemblies [68], GRCh37/hg19 and GRCh38/hg38, of which only the former is natively supported by the integrated tools. To obtain results in a time-efficient manner, we merged pre-calculated files for all the prediction tools into a single database file indexed with Tabix [69] to avoid any need for multiple queries per analyzed variant. An estimated execution time is provided for each user submission based on the number of evaluated variants and the predicted time demands of jobs already waiting in the queue. Raw scores produced by integrated tools and their PredictSNP2 consensus values are transformed onto a single scale ranging from 0 to 99%, corresponding to observed accuracies measured against the testing subsets of individual categories of variants [33]. On the output page (Fig 6), the predictions of individual tools and their consensus are complemented with their confidence scores and are reported together with links to the relevant databases and on-lines services. The user can download the output in human- and machine-readable formats as PDF and VCF files, respectively. Since we found that protein-based predictors could provide improved performance for missense variants, we also added an interlink to the PredictSNP1 web server that enables the user to obtain predictions with these tools for any selected missense variant.

## Availability and Future Directions

To the best of our knowledge, PredictSNP2 represents the first unified platform for nucleotide-based predictions of deleterious variants. This tool is freely available to the scientific and medical community at http://loschmidt.chemi.muni.cz/predictsnp2. The developed datasets (S1–S5 Datasets) and user guide (S2 Text) are also available from the website.

In future, scores for all missense variants will be pre-calculated with the six protein-based tools used in PredictSNP1 to allow instant access to their results. We also plan to assess new tools for predicting the effect of nucleotide variants as they emerge, and will consider integrating such tools into the platform based on the results of these evaluations.

## Supporting Information

S1 Dataset Dataset of nucleotide variants associated with Mendelian diseases. (XLSX)

S2 Dataset Dataset of nucleotide variants associated with complex diseases. (XLSX)

S3 Dataset Dataset of nucleotide variants associated with somatic cancers. (XLSX)

S4 Dataset Dataset of amino acid variants associated with Mendelian diseases. (XLSX)

S5 Dataset Dataset of amino acid variants associated with somatic cancers. (XLSX)

S1 Fig Receiver operating characteristic curves of prediction tools and their consensuses evaluated using the dataset of variants associated with Mendelian diseases. (A) Training and (B) testing subsets of all investigated categories.
(TIF)

S2 Fig Workflow diagram describing the construction of the datasets composed of variants related to complex diseases and somatic cancers. The datasets were prepared by combining deleterious variants from the GWAS catalog or the COSMIC database with neutral variants from the VariSNP database. The resulting datasets were then divided into independent training and testing subsets for each individual category of variants. N/A indicates that not enough variants were assigned to the category to enable the performance evaluation. See S9 and S10 Tables for particular numbers of variants.
(TIF)

S3 Fig Numbers of disease-associated variants overlapping among the three constructed datasets. (TIF)

S4 Fig Performance of nucleotide-based and protein-based prediction tools and their consensuses evaluated using the dataset of variants associated with somatic cancers. (A) Observed normalized accuracy and (B) area under the receiver operating characteristic curve (AUC) values are shown as blue and red bars for nucleotide- and protein-based tools and their consensuses, respectively. The horizontal dashed lines represent average performance values for each tool type.
(TIF)

S1 Table Principles and training datasets of six evaluated prediction tools. (PDF)

S2 Table Description of eight databases and on-line services employed within PredictSNP2 framework. (PDF)

S3 Table Effect of general and category-optimal thresholds on accuracies of six individual prediction tools evaluated using the testing subset of variants associated with Mendelian diseases. (PDF)

S4 Table Performance of six individual prediction tools employing category-optimal thresholds for individual variant categories evaluated using the Mendelian diseases dataset. (PDF)

S5 Table Pairwise correlation of raw scores of the five best-performing prediction tools within the individual categories of variants evaluated using the Mendelian diseases dataset. (PDF)

S6 Table Pairwise correlation of binary predictions of the five best-performing prediction tools within the individual categories of variants evaluated using the Mendelian diseases dataset. (PDF)

S7 Table Performance of the developed PredictSNP2 consensus scores evaluated using the Mendelian diseases dataset. (PDF)

S8 Table Performance of nucleotide- and protein-based prediction tools compared using the Mendelian diseases and cancer datasets. (PDF)

S9 Table Performance of the five best-performing prediction tools employing category-optimal thresholds for individual variant categories evaluated using the complex diseases dataset. (PDF)

S10 Table Performance of the five best-performing prediction tools employing category-optimal thresholds for individual variant categories evaluated using the cancer dataset. (PDF)

S1 Text Description of the performance evaluation metrics employed in this study. (PDF)

S2 Text PredictSNP2 user guide. (PDF)
