# Supplementary figures and images for: PredictSNP2: A Unified Platform for Accurately Evaluating SNP Effects by Exploiting the Different Characteristics of Variants in Distinct Genomic Regions
Source: PLoS Comput Biol. 2016 May 25;12(5):e1004962. doi: 10.1371/journal.pcbi.1004962 (PMC4880439; doi:10.1371/journal.pcbi.1004962)

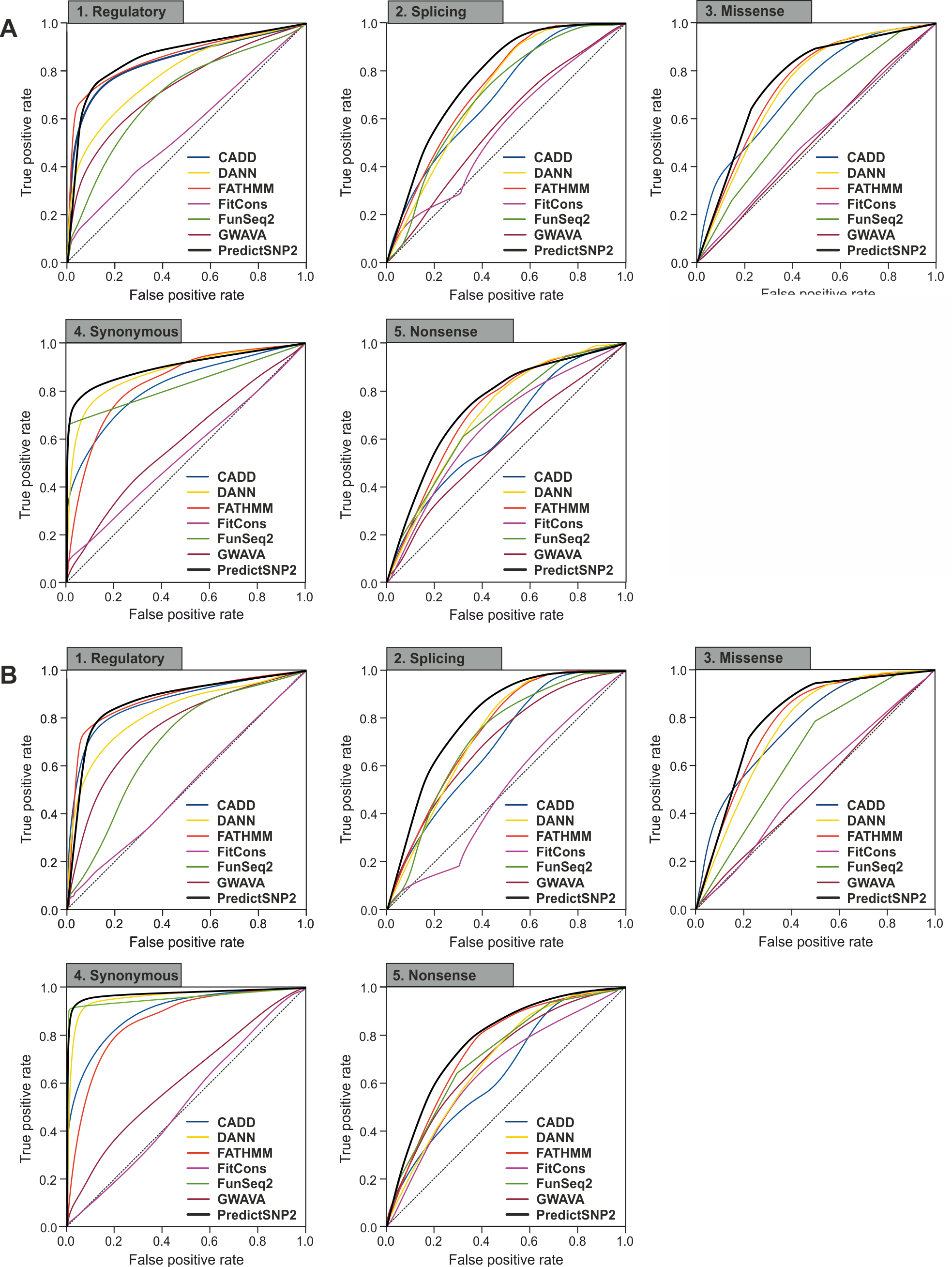

Supplement: S1 Fig — (A) Training and (B) testing subsets of all investigated categories. (TIF) [file pcbi.1004962.s006.tif]

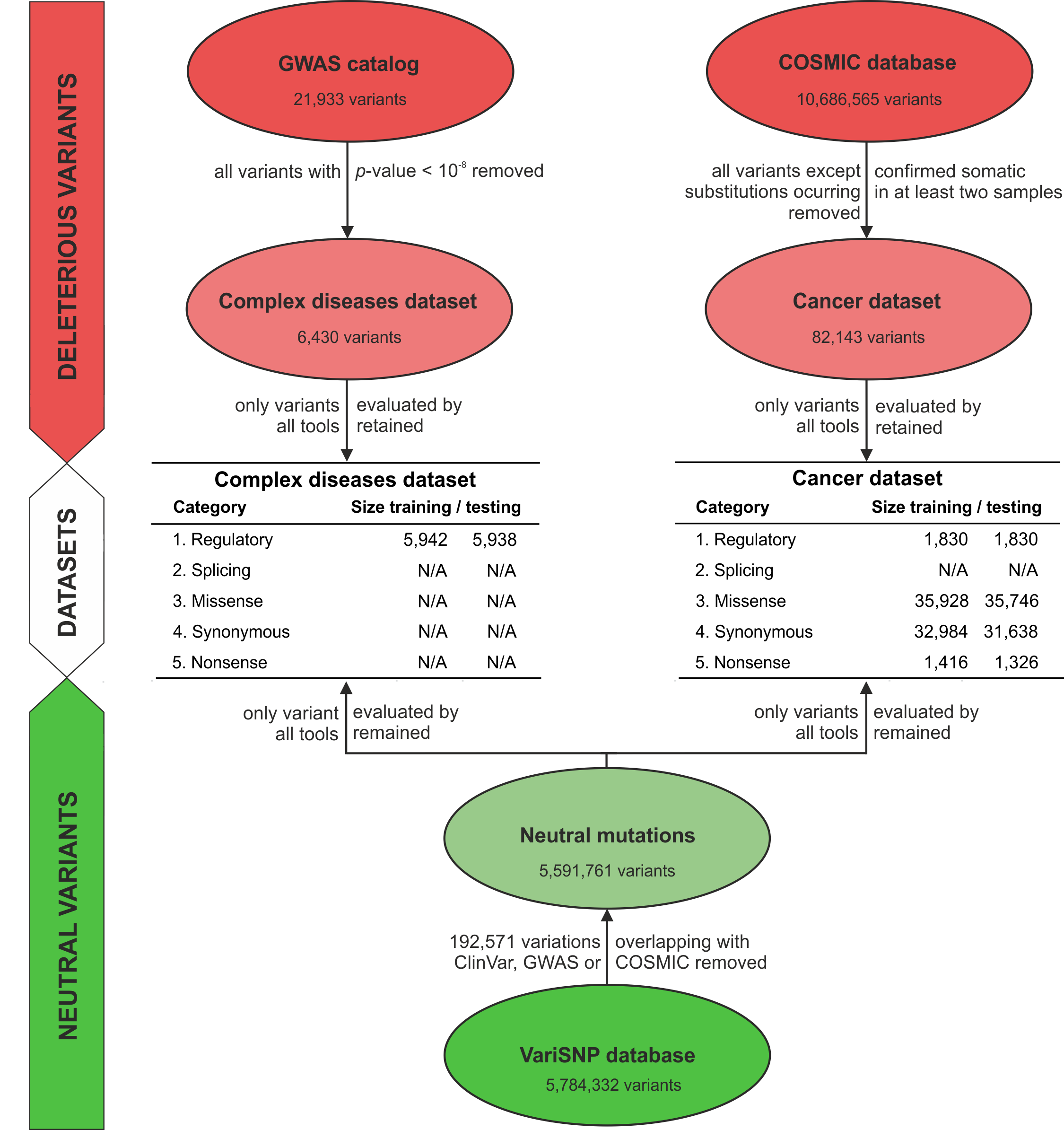

Supplement: S2 Fig — The datasets were prepared by combining deleterious variants from the GWAS catalog or the COSMIC database with neutral variants from the VariSNP database. The resulting datasets were then divided into independent training and testing subsets for each individual category of variants. N/A indicates that not enough variants were assigned to the category to enable the performance evaluation. See S9 and S10 Tables for particular numbers of variants. (TIF) [file pcbi.1004962.s007.tif]

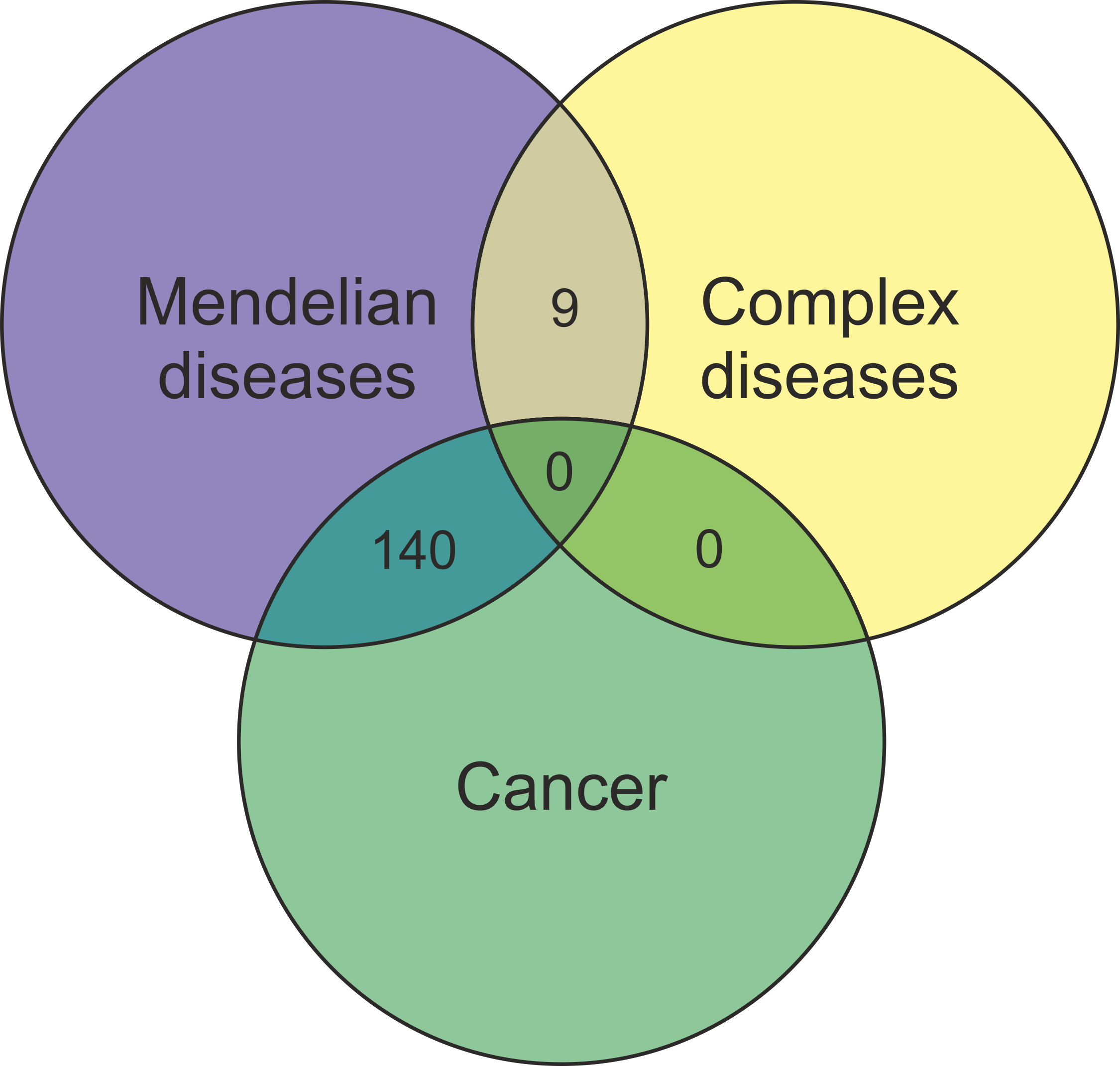

Supplement: S3 Fig — (TIF) [file pcbi.1004962.s008.tif]

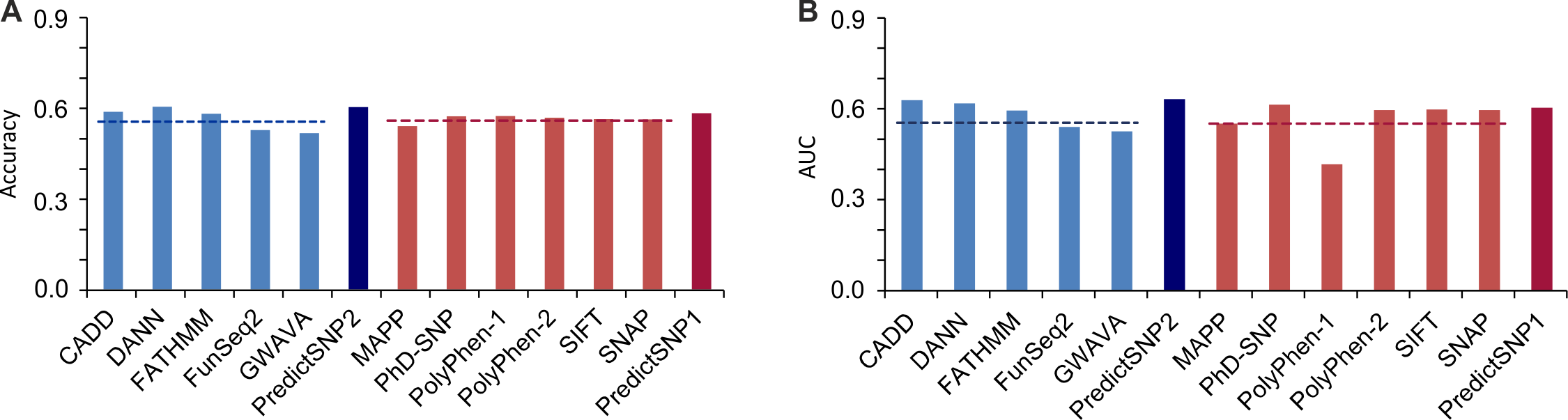

Supplement: S4 Fig — (A) Observed normalized accuracy and (B) area under the receiver operating characteristic curve (AUC) values are shown as blue and red bars for nucleotide- and protein-based tools and their consensuses, respectively. The horizontal dashed lines represent average performance values for each tool type. (TIF) [file pcbi.1004962.s009.tif]
