# Supplementary material for: PredictSNP2: A Unified Platform for Accurately Evaluating SNP Effects by Exploiting the Different Characteristics of Variants in Distinct Genomic Regions
Source: PLoS Comput Biol. 2016 May 25;12(5):e1004962. doi: 10.1371/journal.pcbi.1004962 (PMC4880439; doi:10.1371/journal.pcbi.1004962)
Supplement: S1 Table — (PDF) [file pcbi.1004962.s010.pdf]

S1 Table. Principles and training datasets of six evaluated prediction tools.

Tool name	Version	Principle	Training dataset	Reference
CADD	1.2	Logistic regression	High-frequency human-derived variants<br>14.7 million observed / 14.7 million simulated	[24]
DANN	2014-11-14	Deep neural network	High-frequency human-derived variants<br>14.7 million observed / 14.7 million simulated	[26]
FATHMM	2015-02-25	Support vector machine	HGMD (heritable germline), 1000 Genome (MAF ≥ 1%)<br>12,438 deleterious / 24,064 neutral	[27]
FitCons	1.01<br>Cell type: H1 hESC	Evolutionary fingerprints	No training dataset	[28]
FunSeq2	2.1.2	Weighted scoring system	No training dataset	[32]
GWAVA	1.0<br>Model: region	Random forest	HGMD (regulatory), 1000 Genome (MAF ≥ 1%)<br>1,614 deleterious / 5,027 neutral	[25]

CADD – Combined Annotation Dependent Depletion, DANN – Deleterious Annotation of Genetic Variants using Neural Networks, FATHMM – Functional Analysis through Hidden Markov Models, FitCons – Fitness Consequences of Functional Annotation, GWAVA – Genome-Wide Annotation of Variants, hESC – human embryonic stem cells, HGMD – The Human Gene Mutation Database, MAF – Minor allele frequency
