# Supplementary material for: PredictSNP2: A Unified Platform for Accurately Evaluating SNP Effects by Exploiting the Different Characteristics of Variants in Distinct Genomic Regions
Source: PLoS Comput Biol. 2016 May 25;12(5):e1004962. doi: 10.1371/journal.pcbi.1004962 (PMC4880439; doi:10.1371/journal.pcbi.1004962)
Supplement: S2 Table — (PDF) [file pcbi.1004962.s011.pdf]

S2 Table. Description of eight databases and on-line services employed within PredictSNP2 framework.

Tool resource	Description	Content	Reference
ClinVar	Database of variations containing the interpretations of the relationship to human health and the evidence supporting each interpretation.	Variations: 158,850 [September 2015]	[35]
dbSNP	Database of short genetic variations	Variations: 149,735,377 [build 144]	[46]
Ensembl Genome browser	Genome browser providing an interface to display the information from various biological databases for selected position in the genome	-	[52]
GenBank	Database of all publicly available nucleotide sequences and their protein translations	-	[50]
HaploReg	Database of annotations of variations on haplotype blocks, such as predicted chromatin state, sequence conservation across mammals, the effect on regulatory motif and the effect of expression	52,053,803	[48]
OMIM	Database of variations and genes associated with inherited disorders	Variations: 24,367 [September 2015]	[47]
RegulomeDB	Database of annotations of variations in the intergenic regions using gene expression, ENCODE and data from published articles.	61,397,379	[49]
UCSC Genome browser	Genome browser providing an interface to display the information from various biological databases for selected position in the genome	-	[51]
