# Supplementary material for: PredictSNP2: A Unified Platform for Accurately Evaluating SNP Effects by Exploiting the Different Characteristics of Variants in Distinct Genomic Regions
Source: PLoS Comput Biol. 2016 May 25;12(5):e1004962. doi: 10.1371/journal.pcbi.1004962 (PMC4880439; doi:10.1371/journal.pcbi.1004962)
Supplement: S3 Table — (PDF) [file pcbi.1004962.s012.pdf]

S3 Table. Effects of general and category-optimal thresholds on accuracies of six individual prediction tools evaluated using the testing subset of variants associated with Mendelian diseases.

Performance metrics	Category	# of variants	CADD		DANN		FATHMM		FitCons		FunSeq2		GWAVA	
			optimal	general	optimal	general	optimal	general [a]	optimal	general	optimal	general	optimal	general [a]
**Accuracy**	1. Regulatory	358	0.824	0.654	0.763	0.517	0.821	0.838	0.517	0.503	0.662	0.528	0.701	0.690
	2. Splicing	1,582	0.643	0.642	0.688	0.682	0.692	0.629	0.551	0.531	0.689	0.506	0.626	0.632
	3. Missense	2,692	0.684	0.682	0.726	0.726	0.744	0.676	0.502	0.499	0.644	0.644	0.507	0.507
	4. Synonymous	816	0.825	0.825	0.945	0.826	0.808	0.754	0.496	0.500	0.957	0.886	0.586	0.592
	5. Nonsense	1,068	0.619	0.614	0.653	0.645	0.710	0.699	0.619	0.508	0.673	0.625	0.630	0.607
**Threshold**	1. Regulatory	358	8.698	17.33	0.753	0.993	0.230	0.500	0.122	0.107	0.882	2.012	0.450	0.500
	2. Splicing	1,582	18.09	17.33	0.979	0.993	0.963	0.500	0.081	0.107	0.835	2.012	0.353	0.500
	3. Missense	2,692	18.91	17.33	0.993	0.993	0.897	0.500	0.778	0.107	2.000	2.012	0.245	0.500
	4. Synonymous	816	15.63	17.33	0.889	0.993	0.787	0.500	0.731	0.107	0.977	2.012	0.470	0.500
	5. Nonsense	1,068	16.54	17.33	0.994	0.993	0.723	0.500	0.590	0.107	3.000	2.012	0.453	0.500

[a] These general thresholds were derived already during the development of the tools.
