# Supplementary material for: PredictSNP2: A Unified Platform for Accurately Evaluating SNP Effects by Exploiting the Different Characteristics of Variants in Distinct Genomic Regions
Source: PLoS Comput Biol. 2016 May 25;12(5):e1004962. doi: 10.1371/journal.pcbi.1004962 (PMC4880439; doi:10.1371/journal.pcbi.1004962)
Supplement: S4 Table — (PDF) [file pcbi.1004962.s013.pdf]

S4 Table. Performance of six individual prediction tools employing category-optimal thresholds for individual variant categories evaluated using the Mendelian diseases dataset.

Performance metrics	Category	CADD		DANN		FATHMM		FitCons		FunSeq2		GWAVA	
		train	test	train	test	train	test	train	test	train	test	train	test
**Accuracy**	1. Regulatory	0.807	0.824	0.726	0.763	0.819	0.821	0.553	0.517	0.679	0.662	0.686	0.701
	2. Splicing	0.644	0.643	0.682	0.688	0.689	0.692	0.588	0.551	0.671	0.689	0.568	0.626
	3. Missense	0.657	0.684	0.699	0.726	0.708	0.744	0.502	0.502	0.604	0.644	0.513	0.507
	4. Synonymous	0.763	0.825	0.840	0.945	0.790	0.808	0.509	0.496	0.828	0.957	0.586	0.586
	5. Nonsense	0.596	0.619	0.676	0.653	0.691	0.710	0.631	0.619	0.644	0.673	0.566	0.630
**Matthews correlation coefficient**	1. Regulatory	0.618	0.648	0.459	0.526	0.638	0.646	0.123	0.039	0.358	0.324	0.388	0.410
	2. Splicing	0.320	0.318	0.409	0.424	0.410	0.413	0.184	0.105	0.352	0.395	0.145	0.294
	3. Missense	0.323	0.380	0.403	0.462	0.419	0.496	0.014	0.017	0.212	0.300	0.036	0.017
	4. Synonymous	0.527	0.653	0.688	0.890	0.580	0.615	0.032	0.013	0.693	0.917	0.183	0.182
	5. Nonsense	0.219	0.280	0.361	0.309	0.387	0.423	0.273	0.248	0.289	0.347	0.144	0.277
**AUC** [a]	1. Regulatory	0.846	0.875	0.791	0.831	0.864	0.886	0.553	0.515	0.704	0.696	0.735	0.763
	2. Splicing	0.699	0.689	0.718	0.737	0.738	0.743	0.556	0.494	0.700	0.716	0.573	0.695
	3. Missense	0.729	0.771	0.736	0.763	0.749	0.787	0.519	0.528	0.631	0.658	0.513	0.507
	4. Synonymous	0.822	0.900	0.884	0.962	0.838	0.864	0.537	0.513	0.829	0.959	0.587	0.610
	5. Nonsense	0.630	0.650	0.704	0.691	0.721	0.746	0.649	0.649	0.686	0.719	0.576	0.701
**Sensitivity**	1. Regulatory	0.746	0.816	0.644	0.743	0.814	0.872	0.299	0.263	0.655	0.654	0.542	0.603
	2. Splicing	0.864	0.860	0.910	0.920	0.885	0.879	0.742	0.685	0.794	0.836	0.751	0.884
	3. Missense	0.771	0.812	0.773	0.829	0.764	0.831	0.017	0.021	0.707	0.788	0.860	0.825
	4. Synonymous	0.804	0.877	0.760	0.936	0.770	0.809	0.086	0.083	0.665	0.922	0.411	0.419
	5. Nonsense	0.841	0.884	0.785	0.730	0.773	0.777	0.770	0.760	0.609	0.640	0.361	0.461
**Specificity**	1. Regulatory	0.867	0.832	0.809	0.782	0.824	0.771	0.807	0.771	0.703	0.670	0.830	0.799
	2. Splicing	0.423	0.427	0.454	0.455	0.492	0.504	0.433	0.416	0.548	0.542	0.384	0.368
	3. Missense	0.544	0.556	0.626	0.623	0.652	0.658	0.986	0.984	0.501	0.500	0.165	0.188
	4. Synonymous	0.721	0.772	0.919	0.953	0.809	0.806	0.932	0.909	0.990	0.993	0.760	0.752
	5. Nonsense	0.350	0.354	0.567	0.575	0.609	0.642	0.493	0.478	0.680	0.706	0.770	0.800
**Precision**	1. Regulatory	0.849	0.830	0.771	0.773	0.822	0.792	0.608	0.534	0.688	0.665	0.761	0.750
	2. Splicing	0.600	0.600	0.625	0.628	0.636	0.639	0.567	0.540	0.637	0.646	0.549	0.583
	3. Missense	0.628	0.646	0.674	0.687	0.687	0.708	0.555	0.560	0.586	0.612	0.508	0.504
	4. Synonymous	0.743	0.794	0.904	0.953	0.802	0.807	0.556	0.479	0.986	0.992	0.632	0.629
	5. Nonsense	0.564	0.578	0.645	0.632	0.664	0.685	0.603	0.593	0.655	0.685	0.611	0.697
**NPV** [b]	1. Regulatory	0.774	0.819	0.694	0.753	0.816	0.857	0.535	0.511	0.671	0.659	0.644	0.668
	2. Splicing	0.757	0.753	0.835	0.851	0.811	0.806	0.627	0.569	0.726	0.767	0.607	0.760
	3. Missense	0.703	0.747	0.733	0.785	0.734	0.795	0.501	0.501	0.631	0.702	0.542	0.518
	4. Synonymous	0.787	0.863	0.793	0.937	0.779	0.808	0.505	0.498	0.747	0.927	0.563	0.564
	5. Nonsense	0.688	0.753	0.725	0.681	0.729	0.742	0.681	0.666	0.635	0.663	0.547	0.597
**# of variants**	1. Regulatory	1,056	358	1,056	358	1,056	358	1,056	358	1,056	358	1,056	358
	2. Splicing	1,998	1,582	1,998	1,582	1,998	1,582	1,998	1,582	1,998	1,582	1,998	1,582
	3. Missense	14,024	2,692	14,024	2,692	14,024	2,692	14,024	2,692	14,024	2,692	14,024	2,692
	4. Synonymous	818	816	818	816	818	816	818	816	818	816	818	816
	5. Nonsense	1,068	1,068	1,068	1,068	1,068	1,068	1,068	1,068	1,068	1,068	1,068	1,068

[a] Area under the receiver operating characterics curve.

[b] Negative predictive value.
