# Supplementary material for: PredictSNP2: A Unified Platform for Accurately Evaluating SNP Effects by Exploiting the Different Characteristics of Variants in Distinct Genomic Regions
Source: PLoS Comput Biol. 2016 May 25;12(5):e1004962. doi: 10.1371/journal.pcbi.1004962 (PMC4880439; doi:10.1371/journal.pcbi.1004962)
Supplement: S5 Table — (PDF) [file pcbi.1004962.s014.pdf]

S5 Table. Pairwise correlation of raw scores of the five best-performing prediction tools for the individual variant categories evaluated using the Mendelian diseases dataset.

Category		Spearman correlation coefficient				
		CADD	DANN	FATHMM	GWAVA	FunSeq2
1. Regulatory	CADD	-				
	DANN	0.848	-			
	FATHMM	0.851	0.732	-		
	GWAVA	0.524	0.461	0.516	-	
	FunSeq2	0.460	0.378	0.438	0.352	-
2. Splicing	CADD	-				
	DANN	0.629	-			
	FATHMM	0.612	0.693	-		
	GWAVA	0.295	0.351	0.399	-	
	FunSeq2	0.140	0.260	0.193	0.277	-
3. Missense	CADD	-				
	DANN	0.615	-			
	FATHMM	0.632	0.527	-		
	GWAVA	0.148	0.081	0.215	-	
	FunSeq2	0.345	0.267	0.414	0.247	-
4. Synonymous	CADD	-				
	DANN	0.794	-			
	FATHMM	0.643	0.687	-		
	GWAVA	0.224	0.194	0.311	-	
	FunSeq2	0.679	0.809	0.657	0.241	-
5. Nonsense	CADD	-				
	DANN	0.410	-			
	FATHMM	0.438	0.620	-		
	GWAVA	0.151	0.308	0.416	-	
	FunSeq2	0.293	0.420	0.483	0.392	-
