# Supplementary material for: PredictSNP2: A Unified Platform for Accurately Evaluating SNP Effects by Exploiting the Different Characteristics of Variants in Distinct Genomic Regions
Source: PLoS Comput Biol. 2016 May 25;12(5):e1004962. doi: 10.1371/journal.pcbi.1004962 (PMC4880439; doi:10.1371/journal.pcbi.1004962)
Supplement: S6 Table — (PDF) [file pcbi.1004962.s015.pdf]

S6 Table. Pairwise correlation of binary predictions of the five best-performing prediction tools for the individual variant categories evaluated using the Mendelian diseases dataset.

Category		Spearman correlation coefficient				
		**CADD**	**DANN**	**FATHMM**	**GWAVA**	**FunSeq2**
**1. Regulatory**	**CADD**	-	0.131	0.112	0.089	0.101
	**DANN**	0.718	-	0.092	0.095	0.098
	**FATHMM**	0.757	0.676	-	0.075	0.117
	**GWAVA**	0.615	0.559	0.598	-	0.148
	**FunSeq2**	0.587	0.522	0.601	0.511	-
**2. Splicing**	**CADD**	-	0.238	0.227	0.217	0.146
	**DANN**	0.570	-	0.236	0.215	0.159
	**FATHMM**	0.562	0.615	-	0.203	0.127
	**GWAVA**	0.486	0.528	0.520	-	0.178
	**FunSeq2**	0.479	0.535	0.508	0.493	-
**3. Missense**	**CADD**	-	0.175	0.165	0.215	0.172
	**DANN**	0.584	-	0.136	0.178	0.136
	**FATHMM**	0.593	0.606	-	0.178	0.155
	**GWAVA**	0.406	0.411	0.429	-	0.247
	**FunSeq2**	0.499	0.505	0.542	0.397	-
**4. Synonymous**	**CADD**	-	0.034	0.064	0.080	0.018
	**DANN**	0.804	-	0.023	0.029	0.026
	**FATHMM**	0.696	0.776	-	0.112	0.016
	**GWAVA**	0.490	0.560	0.505	-	0.031
	**FunSeq2**	0.800	0.928	0.781	0.574	-
**5. Nonsense**	**CADD**	-	0.211	0.184	0.107	0.141
	**DANN**	0.482	-	0.211	0.148	0.166
	**FATHMM**	0.512	0.573	-	0.140	0.148
	**GWAVA**	0.356	0.431	0.479	-	0.165
	**FunSeq2**	0.434	0.492	0.531	0.468	-

Lower left triangle: ratio of cases predicted correctly by both tools.

Upper right triangle: ratio of cases predicted incorrectly by both tools.
