# Supplementary material for: PredictSNP2: A Unified Platform for Accurately Evaluating SNP Effects by Exploiting the Different Characteristics of Variants in Distinct Genomic Regions
Source: PLoS Comput Biol. 2016 May 25;12(5):e1004962. doi: 10.1371/journal.pcbi.1004962 (PMC4880439; doi:10.1371/journal.pcbi.1004962)
Supplement: S7 Table — (PDF) [file pcbi.1004962.s016.pdf]

S7 Table. Performance of the developed PredictSNP2 consensus scores evaluated using the Mendelian diseases dataset.

Performance metrics	Category	PSNP5		PSNP4		PSNP3		PSNP2	
		train	test	train	test	train	test	train	test
**Accuracy**	1. Regulatory	0.822	0.844	0.827	0.852	0.822	0.855	0.818	0.852
	2. Splicing	0.723	0.745	0.742	0.737	0.747	0.745	0.696	0.697
	3. Missense	0.721	0.761	0.724	0.764	0.730	0.764	0.734	0.773
	4. Synonymous	0.868	0.945	0.870	0.958	0.875	0.957	0.840	0.947
	5. Nonsense	0.722	0.724	0.718	0.707	0.711	0.710	0.701	0.698
**Matthews correlation coefficient**	1. Regulatory	0.656	0.689	0.660	0.704	0.657	0.712	0.647	0.705
	2. Splicing	0.446	0.493	0.492	0.482	0.508	0.505	0.440	0.441
	3. Missense	0.447	0.533	0.457	0.545	0.461	0.531	0.469	0.550
	4. Synonymous	0.741	0.890	0.756	0.917	0.761	0.914	0.687	0.895
	5. Nonsense	0.444	0.448	0.437	0.415	0.421	0.420	0.405	0.396
**AUC** [a]	1. Regulatory	0.868	0.884	0.871	0.888	0.863	0.874	0.858	0.866
	2. Splicing	0.776	0.803	0.790	0.798	0.793	0.801	0.717	0.723
	3. Missense	0.758	0.803	0.767	0.817	0.765	0.806	0.765	0.804
	4. Synonymous	0.901	0.974	0.904	0.977	0.905	0.978	0.865	0.972
	5. Nonsense	0.743	0.777	0.748	0.763	0.751	0.754	0.729	0.722
**Sensitivity**	1. Regulatory	0.725	0.804	0.758	0.832	0.722	0.810	0.727	0.821
	2. Splicing	0.754	0.799	0.833	0.826	0.865	0.866	0.922	0.920
	3. Missense	0.795	0.863	0.817	0.889	0.745	0.813	0.758	0.833
	4. Synonymous	0.812	0.953	0.770	0.939	0.795	0.951	0.765	0.949
	5. Nonsense	0.727	0.727	0.699	0.670	0.719	0.695	0.757	0.730
**Specificity**	1. Regulatory	0.919	0.883	0.896	0.872	0.922	0.899	0.909	0.883
	2. Splicing	0.692	0.692	0.651	0.649	0.629	0.625	0.470	0.474
	3. Missense	0.647	0.660	0.632	0.638	0.716	0.716	0.710	0.713
	4. Synonymous	0.924	0.936	0.971	0.978	0.956	0.963	0.914	0.946
	5. Nonsense	0.717	0.721	0.738	0.743	0.702	0.725	0.646	0.665
**Precision**	1. Regulatory	0.899	0.873	0.879	0.866	0.903	0.890	0.889	0.875
	2. Splicing	0.710	0.721	0.704	0.701	0.700	0.698	0.635	0.636
	3. Missense	0.693	0.717	0.689	0.711	0.724	0.741	0.723	0.744
	4. Synonymous	0.915	0.937	0.963	0.977	0.948	0.963	0.899	0.946
	5. Nonsense	0.720	0.723	0.727	0.723	0.707	0.716	0.681	0.685
**NPV** [b]	1. Regulatory	0.770	0.819	0.787	0.839	0.768	0.826	0.769	0.832
	2. Splicing	0.737	0.775	0.796	0.788	0.823	0.823	0.858	0.856
	3. Missense	0.759	0.828	0.775	0.852	0.737	0.793	0.746	0.810
	4. Synonymous	0.831	0.953	0.809	0.941	0.823	0.952	0.796	0.948
	5. Nonsense	0.724	0.725	0.710	0.693	0.714	0.704	0.726	0.711
**# of variants**	1. Regulatory	1,056	358	1,056	358	1,056	358	1,056	358
	2. Splicing	1,998	1,582	1,998	1,582	1,998	1,582	1,998	1,582
	3. Missense	14,024	2,692	14,024	2,692	14,024	2,692	14,024	2,692
	4. Synonymous	818	816	818	816	818	816	818	816
	5. Nonsense	1,068	1,068	1,068	1,068	1,068	1,068	1,068	1,068
**Tools included in the consensus** [c]	1. Regulatory	FA, CA, DA, GW, FU		FA, CA, DA, GW		FA, CA, DA		FA, CA	
	2. Splicing	FA, DA, FU, CA, GW		FA, DA, FU, CA		FA, DA, FU		FA, DA	
	3. Missense	FA, DA, CA, FU, GW		FA, DA, CA, FU		FA, DA, CA		FA, DA	
	4. Synonymous	DA, FU, FA, CA, GW		DA, FU, FA, CA		DA, FU, FA		DA, FU	
	5. Nonsense	FA, DA, FU, CA, GW		FA, DA, FU, CA		FA, DA, FU		FA, DA	

[a] Area under the receiver operating characteristic curve.

[b] Negative predictive value.

[c] Tools are ordered by their accuracies on training dataset. Abbreviations: CA – CADD, DA – DANN, FA – FATHMM, FU – FunSeq2, GW – GWAVA.
