# Supplementary material for: PredictSNP2: A Unified Platform for Accurately Evaluating SNP Effects by Exploiting the Different Characteristics of Variants in Distinct Genomic Regions
Source: PLoS Comput Biol. 2016 May 25;12(5):e1004962. doi: 10.1371/journal.pcbi.1004962 (PMC4880439; doi:10.1371/journal.pcbi.1004962)
Supplement: S8 Table — (PDF) [file pcbi.1004962.s017.pdf]

S8 Table. Performance of nucleotide- and protein-based prediction tools compared using the Mendelian diseases and cancer datasets.

		Mendelian diseases dataset			Cancer dataset		
**Tool category**	**Tool name**	**ACC [a]**	**MCC [b]**	**AUC [c]**	**ACC [a]**	**MCC [b]**	**AUC [c]**
**Nucleotide-based**	CADD	0.684	0.380	0.771	0.591	0.182	0.623
	DANN	0.726	0.462	0.763	0.608	0.219	0.615
	FATHMM	0.744	0.496	0.787	0.585	0.174	0.595
	FunSeq2	0.644	0.300	0.658	0.530	0.060	0.538
	GWAVA	0.507	0.017	0.507	0.520	0.040	0.527
	**PredictSNP2**	**0.773**	**0.550**	**0.804**	**0.606**	**0.218**	**0.624**
**Protein-based**	MAPP	0.659	0.322	0.683	0.544	0.094	0.554
	PhD-SNP	0.760	0.521	0.826	0.576	0.171	0.612
	PolyPhen-1	0.714	0.427	0.730	0.577	0.160	0.417
	PolyPhen-2	0.687	0.389	0.725	0.571	0.143	0.592
	SIFT	0.730	0.473	0.779	0.567	0.134	0.594
	SNAP	0.691	0.386	0.752	0.566	0.142	0.592
	**PredictSNP1**	**0.755**	**0.510**	**0.803**	**0.586**	**0.183**	**0.605**

[a] Normalized accuracy.
[b] Matthews correlation coefficient.
[c] Area under the receiver operating characteristic curve.
