# Supplementary material for: PredictSNP2: A Unified Platform for Accurately Evaluating SNP Effects by Exploiting the Different Characteristics of Variants in Distinct Genomic Regions
Source: PLoS Comput Biol. 2016 May 25;12(5):e1004962. doi: 10.1371/journal.pcbi.1004962 (PMC4880439; doi:10.1371/journal.pcbi.1004962)
Supplement: S9 Table — (PDF) [file pcbi.1004962.s018.pdf]

S9 Table. Performance of the five best-performing prediction tools employing category-optimal thresholds for individual variant categories evaluated using the complex diseases dataset.

Performance metrics	Category	CADD		DANN		FATHMM		FunSeq2		GWAVA	
		train	test	train	test	train	test	train	test	train	test
**Accuracy**	1. Regulatory	0.507	0.494	0.501	0.499	0.500	0.499	0.504	0.498	0.502	0.502
	2. Splicing	-[a]	-[a]	-[a]	-[a]	-[a]	-[a]	-[a]	-[a]	-[a]	-[a]
	3. Missense	-[a]	-[a]	-[a]	-[a]	-[a]	-[a]	-[a]	-[a]	-[a]	-[a]
	4. Synonymous	-[a]	-[a]	-[a]	-[a]	-[a]	-[a]	-[a]	-[a]	-[a]	-[a]
	5. Nonsense	-[a]	-[a]	-[a]	-[a]	-[a]	-[a]	-[a]	-[a]	-[a]	-[a]
**Matthews correlation coefficient**	1. Regulatory	0.017	-0.014	0.014	-0.009	0.003	-0.009	0.027	-0.014	0.021	0.017
	2. Splicing	-[a]	-[a]	-[a]	-[a]	-[a]	-[a]	-[a]	-[a]	-[a]	-[a]
	3. Missense	-[a]	-[a]	-[a]	-[a]	-[a]	-[a]	-[a]	-[a]	-[a]	-[a]
	4. Synonymous	-[a]	-[a]	-[a]	-[a]	-[a]	-[a]	-[a]	-[a]	-[a]	-[a]
	5. Nonsense	-[a]	-[a]	-[a]	-[a]	-[a]	-[a]	-[a]	-[a]	-[a]	-[a]
**AUC**[b]	1. Regulatory	0.513	0.519	0.533	0.536	0.575	0.578	0.518	0.536	0.536	0.537
	2. Splicing	-[a]	-[a]	-[a]	-[a]	-[a]	-[a]	-[a]	-[a]	-[a]	-[a]
	3. Missense	-[a]	-[a]	-[a]	-[a]	-[a]	-[a]	-[a]	-[a]	-[a]	-[a]
	4. Synonymous	-[a]	-[a]	-[a]	-[a]	-[a]	-[a]	-[a]	-[a]	-[a]	-[a]
	5. Nonsense	-[a]	-[a]	-[a]	-[a]	-[a]	-[a]	-[a]	-[a]	-[a]	-[a]
**Sensitivity**	1. Regulatory	0.755	0.747	0.994	0.994	0.019	0.016	0.030	0.028	0.009	0.011
	2. Splicing	-[a]	-[a]	-[a]	-[a]	-[a]	-[a]	-[a]	-[a]	-[a]	-[a]
	3. Missense	-[a]	-[a]	-[a]	-[a]	-[a]	-[a]	-[a]	-[a]	-[a]	-[a]
	4. Synonymous	-[a]	-[a]	-[a]	-[a]	-[a]	-[a]	-[a]	-[a]	-[a]	-[a]
	5. Nonsense	-[a]	-[a]	-[a]	-[a]	-[a]	-[a]	-[a]	-[a]	-[a]	-[a]
**Specificity**	1. Regulatory	0.259	0.241	0.008	0.005	0.982	0.982	0.978	0.967	0.994	0.992
	2. Splicing	-[a]	-[a]	-[a]	-[a]	-[a]	-[a]	-[a]	-[a]	-[a]	-[a]
	3. Missense	-[a]	-[a]	-[a]	-[a]	-[a]	-[a]	-[a]	-[a]	-[a]	-[a]
	4. Synonymous	-[a]	-[a]	-[a]	-[a]	-[a]	-[a]	-[a]	-[a]	-[a]	-[a]
	5. Nonsense	-[a]	-[a]	-[a]	-[a]	-[a]	-[a]	-[a]	-[a]	-[a]	-[a]
**Precision**	1. Regulatory	0.505	0.496	0.501	0.500	0.509	0.465	0.582	0.462	0.622	0.586
	2. Splicing	-[a]	-[a]	-[a]	-[a]	-[a]	-[a]	-[a]	-[a]	-[a]	-[a]
	3. Missense	-[a]	-[a]	-[a]	-[a]	-[a]	-[a]	-[a]	-[a]	-[a]	-[a]
	4. Synonymous	-[a]	-[a]	-[a]	-[a]	-[a]	-[a]	-[a]	-[a]	-[a]	-[a]
	5. Nonsense	-[a]	-[a]	-[a]	-[a]	-[a]	-[a]	-[a]	-[a]	-[a]	-[a]
**NPV**[c]	1. Regulatory	0.514	0.488	0.585	0.441	0.500	0.499	0.502	0.499	0.501	0.501
	2. Splicing	-[a]	-[a]	-[a]	-[a]	-[a]	-[a]	-[a]	-[a]	-[a]	-[a]
	3. Missense	-[a]	-[a]	-[a]	-[a]	-[a]	-[a]	-[a]	-[a]	-[a]	-[a]
	4. Synonymous	-[a]	-[a]	-[a]	-[a]	-[a]	-[a]	-[a]	-[a]	-[a]	-[a]
	5. Nonsense	-[a]	-[a]	-[a]	-[a]	-[a]	-[a]	-[a]	-[a]	-[a]	-[a]
**# of variants**	1. Regulatory	5,942	5,938	5,942	5,938	5,941	5,938	5,942	5,938	5,942	5,938
	2. Splicing	4	2	4	2	4	2	4	2	4	2
	3. Missense	16	12	16	12	16	12	16	12	16	12
	4. Synonymous	66	64	66	64	66	64	66	64	66	64
	5. Nonsense	4	2	4	2	4	2	4	2	4	2

[a] Number of variants in this category is too low to report any statistical metrics.
[b] Area under the receiver operating characteristic curve.
[c] Negative predictive value.
