# Supplementary material for: PredictSNP2: A Unified Platform for Accurately Evaluating SNP Effects by Exploiting the Different Characteristics of Variants in Distinct Genomic Regions
Source: PLoS Comput Biol. 2016 May 25;12(5):e1004962. doi: 10.1371/journal.pcbi.1004962 (PMC4880439; doi:10.1371/journal.pcbi.1004962)
Supplement: S10 Table — (PDF) [file pcbi.1004962.s019.pdf]

S10 Table. Performance of the five best-performing prediction tools employing category-optimal thresholds for individual variant categories evaluated using the cancer dataset.

Performance metrics	Category	CADD		DANN		FATHMM		FunSeq2		GWAVA	
		train	test	train	test	train	test	train	test	train	test
**Accuracy**	1. Regulatory	0.599	0.605	0.608	0.591	0.614	0.624	0.663	0.662	0.534	0.519
	2. Splicing	- [a]	- [a]	- [a]	- [a]	- [a]	- [a]	- [a]	- [a]	- [a]	- [a]
	3. Missense	0.588	0.591	0.604	0.608	0.584	0.585	0.526	0.530	0.523	0.520
	4. Synonymous	0.526	0.515	0.522	0.511	0.502	0.501	0.500	0.500	0.500	0.499
	5. Nonsense	0.597	0.590	0.631	0.637	0.648	0.632	0.605	0.620	0.595	0.577
**Matthews correlation coefficient**	1. Regulatory	0.210	0.227	0.236	0.198	0.260	0.285	0.351	0.349	0.089	0.049
	2. Splicing	- [a]	- [a]	- [a]	- [a]	- [a]	- [a]	- [a]	- [a]	- [a]	- [a]
	3. Missense	0.177	0.182	0.211	0.219	0.172	0.173	0.052	0.060	0.047	0.039
	4. Synonymous	0.053	0.030	0.044	0.023	0.009	0.002	0.000	-0.006	0.004	-0.011
	5. Nonsense	0.195	0.180	0.263	0.274	0.295	0.264	0.213	0.243	0.206	0.164
**AUC** [b]	1. Regulatory	0.595	0.599	0.613	0.584	0.625	0.654	0.634	0.640	0.520	0.516
	2. Splicing	- [a]	- [a]	- [a]	- [a]	- [a]	- [a]	- [a]	- [a]	- [a]	- [a]
	3. Missense	0.618	0.623	0.602	0.615	0.593	0.595	0.535	0.538	0.529	0.527
	4. Synonymous	0.524	0.510	0.526	0.519	0.506	0.512	0.499	0.498	0.519	0.529
	5. Nonsense	0.637	0.633	0.648	0.657	0.652	0.647	0.641	0.653	0.621	0.609
**Sensitivity**	1. Regulatory	0.440	0.422	0.407	0.400	0.370	0.377	0.480	0.475	0.212	0.204
	2. Splicing	- [a]	- [a]	- [a]	- [a]	- [a]	- [a]	- [a]	- [a]	- [a]	- [a]
	3. Missense	0.566	0.574	0.517	0.527	0.476	0.483	0.515	0.519	0.574	0.568
	4. Synonymous	0.641	0.602	0.464	0.440	0.065	0.060	0.000	0.000	0.017	0.014
	5. Nonsense	0.572	0.548	0.610	0.587	0.648	0.624	0.507	0.535	0.408	0.403
**Specificity**	1. Regulatory	0.758	0.789	0.810	0.783	0.857	0.871	0.847	0.848	0.856	0.834
	2. Splicing	- [a]	- [a]	- [a]	- [a]	- [a]	- [a]	- [a]	- [a]	- [a]	- [a]
	3. Missense	0.611	0.608	0.691	0.689	0.691	0.687	0.537	0.541	0.472	0.471
	4. Synonymous	0.411	0.428	0.580	0.583	0.939	0.941	1.000	1.000	0.984	0.983
	5. Nonsense	0.623	0.632	0.653	0.686	0.647	0.640	0.702	0.704	0.782	0.751
**Precision**	1. Regulatory	0.646	0.667	0.681	0.648	0.721	0.745	0.758	0.758	0.595	0.552
	2. Splicing	- [a]	- [a]	- [a]	- [a]	- [a]	- [a]	- [a]	- [a]	- [a]	- [a]
	3. Missense	0.592	0.594	0.626	0.629	0.607	0.607	0.527	0.531	0.521	0.518
	4. Synonymous	0.521	0.513	0.525	0.513	0.517	0.504	0.500	0.000	0.515	0.458
	5. Nonsense	0.603	0.598	0.637	0.652	0.647	0.634	0.630	0.644	0.652	0.618
**NPV** [c]	1. Regulatory	0.575	0.577	0.577	0.566	0.576	0.583	0.620	0.618	0.521	0.512
	2. Splicing	- [a]	- [a]	- [a]	- [a]	- [a]	- [a]	- [a]	- [a]	- [a]	- [a]
	3. Missense	0.584	0.588	0.589	0.593	0.569	0.570	0.525	0.529	0.526	0.522
	4. Synonymous	0.534	0.518	0.520	0.510	0.501	0.500	0.500	0.500	0.500	0.499
	5. Nonsense	0.593	0.583	0.626	0.624	0.648	0.630	0.587	0.603	0.569	0.557
**# of variants**	1. Regulatory	1,830	1,830	1,830	1,830	1,830	1,830	1,830	1,830	1,830	1,830
	2. Splicing	12	12	12	12	12	12	12	12	12	12
	3. Missense	35,928	35,746	35,928	35,746	35,928	35,746	35,928	35,746	35,928	35,746
	4. Synonymous	32,984	31,638	32,984	31,638	32,984	31,638	32,984	31,638	32,984	31,638
	5. Nonsense	1,416	1,326	1,416	1,326	1,416	1,326	1,416	1,326	1,416	1,326

[a] Number of variants in this category is too low to report any statistical metrics.

[b] Area under the receiver operating characteristic curve.

[c] Negative predictive value.
