# Supplementary material for: PredictSNP2: A Unified Platform for Accurately Evaluating SNP Effects by Exploiting the Different Characteristics of Variants in Distinct Genomic Regions
Source: PLoS Comput Biol. 2016 May 25;12(5):e1004962. doi: 10.1371/journal.pcbi.1004962 (PMC4880439; doi:10.1371/journal.pcbi.1004962)
Supplement: S1 Text — (PDF) [file pcbi.1004962.s020.pdf]

S1 Text. Description of the performance evaluation metrics employed in this study.

The performance of evaluated tools was assessed using the following measures:

- $Sensitivity = \frac{TP}{TP + FN}$
- $Specificity = \frac{TN}{FP + TN}$
- $Precision = \frac{TP}{TP + FP}$
- $Negative\ predictive\ value\ = \frac{TN}{TN + FN}$
- $Normalized\ accuracy = \frac{1}{2}\left(\frac{TP}{TP + FN} + \frac{TN}{FP + TN}\right)$
- $Matthews\ correlation\ coefficient = \frac{TP \cdot TN - FP \cdot FN}{\sqrt{(TN + FN) \cdot (TN+FP) \cdot (TP+FN) \cdot (TP+FP)}}$

In the equations above, following parameters are used: true positive (TP), true negative (TN), false positive (FP) and false negative (FN) cases. The positive cases represent the deleterious variants, while the negative cases represent the neutral variants. Sensitivity and specificity express the ratio of correctly predicted deleterious and neutral cases, respectively. Precision and negative predictive value represent the conditional probability that the deleterious variants will be classified as deleterious and the neutral variants will be classified as neutral. These four metrics do not provide information about the overall performance and they could be biased by imbalanced ratio between neutral and deleterious parts of the dataset. In contrast, the normalized accuracy and Matthews correlation coefficient (MCC) are capable of correct evaluation of uneven datasets, and therefore are regarded as more significant measures. With the exception of MCC, all metrics are in a range from 0 to 1. MCC spans the interval $\langle -1, +1 \rangle$, where +1 represents the perfect prediction, 0 represents the random prediction and -1 represents the perfect negative correlation of predicted and actual classes. The receiver operating characteristics (ROC) curve is the technique for analysis of the performance of the predictor on different decision thresholds, which shows the general trade-off between the true positive rate and the false positive rate. The area under the ROC curve (AUC) reduces the complex outcome of ROC analysis to a single scalar value. While AUC equal to 1 indicates a perfect correlation, the random prediction is expressed by AUC equal to 0.5. AUC can be interpreted as the probability that a classifier will rank a randomly chosen positive instance higher than a randomly chosen negative one. Therefore, it describes well the ability of classifiers to prioritize variants.
