# Supplementary material for: PredictSNP2: A Unified Platform for Accurately Evaluating SNP Effects by Exploiting the Different Characteristics of Variants in Distinct Genomic Regions
Source: PLoS Comput Biol. 2016 May 25;12(5):e1004962. doi: 10.1371/journal.pcbi.1004962 (PMC4880439; doi:10.1371/journal.pcbi.1004962)
Supplement: S2 Text — (PDF) [file pcbi.1004962.s021.pdf]

PredictSNP2: A unified platform for accurately evaluating SNP effects by exploiting the different characteristics of variants in distinct genomic regions

User guide

Contact:

Loschmidt Laboratories,

Department of Experimental Biology and Research Centre for Toxic Compounds in the Environment,

Faculty of Science, Masaryk University,

Kamenice 5, Bld. A13, 625 00 Brno, Czech Republic

Webpage: http://loschmidt.chemi.muni.cz

E-mail: predictsnp@gmail.com

Recommended Citation:

Bendl, J., Musil, M., Stourac, J., Zendulka, J., Damborsky, J., Brezovsky, J., 2015: **PredictSNP2: A unified platform for accurately evaluating SNP effects by exploiting the different characteristics of variants in distinct genomic regions**. Submitted to publication.

© Copyright 2011-2016 Loschmidt Laboratories, Department of Experimental Biology and Research Centre for Toxic Compounds in the Environment, Faculty of Science, Masaryk University, Brno, Czech Republic

Contents

1. Introduction ____ **4**

2. Input page ____ **5**

3. Output page ____ **7**

4. Example ____ **9**

1. INTRODUCTION

The precise delineation of phenotypically causal variants plays a key role in providing accurate personalized diagnosis, prognosis, and treatment of inherited diseases. Several computational tools have already been developed for this purpose. The PredictSNP2 is a consensus classifier combining five best performing prediction methods to provide more accurate and robust alternative to the predictions delivered by individual integrated tools. The predictions from the computational tools are supplemented by experimental annotations from eight databases. The web server is freely available to the academic community at http://loschmidt.chemi.muni.cz/predictsnp2.

Integrated prediction tools

Tool name	Version	Principle	Training dataset
CADD	1.2	Logistic regression	High-frequency human-derived variants<br>14.7 million observed / 14.7 million simulated
DANN	2014-11-14	Deep neural network	High-frequency human-derived variants<br>14.7 million observed / 14.7 million simulated
FATHMM	2015-02-25	Support vector machine	HGMD (heritable germline), 1000 Genome (MAF ≥ 1%)<br>12,438 deleterious / 24,064 neutral
FunSeq2	2.1.2	Weighted scoring system	No training dataset
GWAVA	1.0<br>model: region	Random forest	HGMD (regulatory), 1000 Genome (MAF ≥ 1%)<br>1,614 deleterious / 5,027 neutral

CADD – Combined Annotation Dependent Depletion, DANN – Deleterious Annotation of Genetic Variants using Neural Networks, FATHMM – Functional Analysis through Hidden Markov Models, GWAVA – Genome-Wide Annotation of Variants, HGMD – The Human Gene Mutation Database, MAF – Minor allele

Referenced databases and on-line services

Tool resource	Description	Content
ClinVar	Database of variations containing the interpretations of the relationship to human health and the evidence supporting each interpretation.	Variations: 158,850<br>[September 2015]
dbSNP	Database of short genetic variations	Variations: 149,735,377<br>[build 144]
Ensembl Genome browser	Genome browser providing an interface to display the information from various biological databases for selected position in the genome	-
GenBank	Database of all publicly available nucleotide sequences and their protein translation	-
HaploReg	Database of annotations of variations on haplotype blocks, such as predicted chromatin state, sequence conservation across mammals, the effect on regulatory motif and the effect of expression	52,053,803
OMIM	Database of variations and genes associated with inherited disorders	Variations: 24,367<br>[September 2015]
RegulomeDB	Database of annotations of variations in the intergenic regions using gene expression, ENCODE and data from published articles.	61,397,379
UCSC Genome browser	Genome browser providing an interface to display the information from various biological databases for selected position in the genome	-

2. INPUT PAGE

The process of submitting the job to PredictSNP server consists of following steps:

1. **INPUT** section
    a. Upload the file or insert the list of genetic variants in one of supported formats - see table below:

Simple	Definition: chromosome,position_start,[position_end],reference_allele,alternative_allele<br>Example – chromosome coordinates: `chr14,51062341,G,C`
VCF	References: VCF format specification, journal article<br>Example – chromosome coordinates: `chr14 51062341 . G C . . .`
GVF	References: GVF format specification, journal article<br>Example – chromosome coordinates: `chr18 . SNV 21118528 21118528 . + . Reference_seq=G;Variant_seq=C`
HGVS	References: HGVS format specification, journal article<br>Example – RefSeq coordinates: NM_000271.4:c.3019G>C

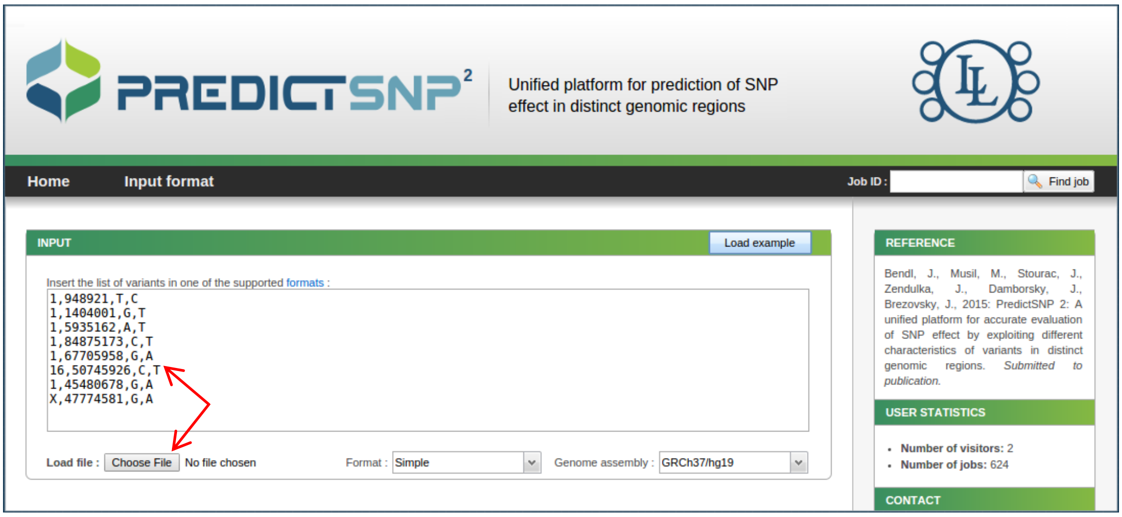

    b. Select *format* and *genome assembly* of your input.

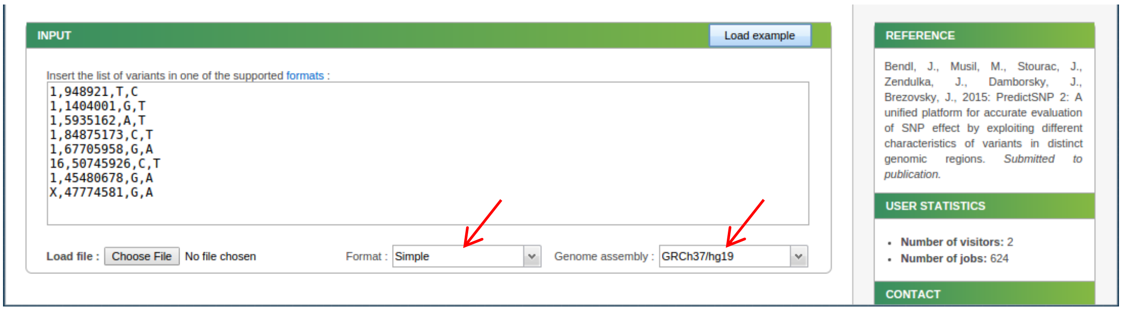

2. **JOB INFO** section (OPTIONALLY): Provide a job title and e-mail address on which the information about the job will be sent. The number of jobs waiting in the queue is reported and waiting time is estimated based on the number of variants already submitted for evaluation.

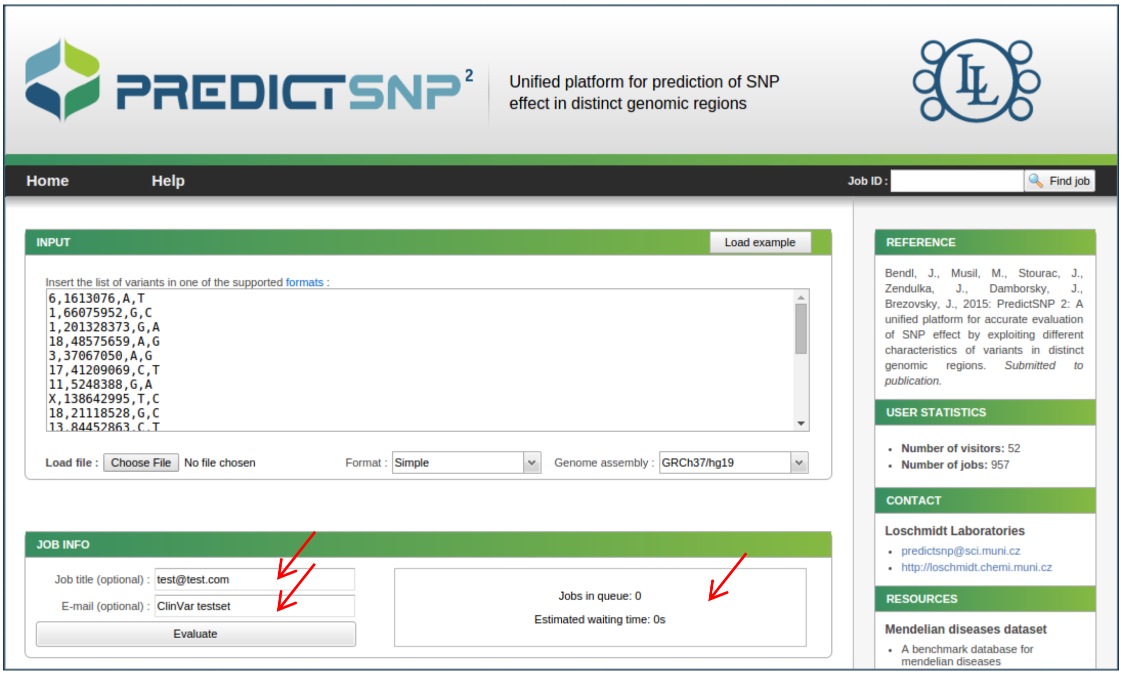
PREDICTSNP 2
Unified platform for prediction of SNP effect in distinct genomic regions
Home
Help
Job ID :
Find job
INPUT
Load example
Insert the list of variants in one of the supported formats :
6,1613076,A,T
1,66075952,G,C
1,201328373,G,A
18,48575659,A,G
3,37067050,A,G
17,41209069,C,T
11,5248388,G,A
X,138642995,T,C
18,21118528,G,C
Load file : Choose File No file chosen
Format : Simple
Genome assembly : GRCh37/hg19
JOB INFO
Job title (optional) : test@test.com
E-mail (optional) : ClinVar testset
Evaluate
Jobs in queue: 0
Estimated waiting time: 0s
REFERENCE
Bendl, J., Musil, M., Stourac, J., Zendulka, J., Damborsky, J., Brezovsky, J., 2015: PredictSNP 2: A unified platform for accurate evaluation of SNP effect by exploiting different characteristics of variants in distinct genomic regions. Submitted to publication.
USER STATISTICS
Number of visitors: 52
Number of jobs: 957
CONTACT
Loschmidt Laboratories
predictsnp@sci.muni.cz
http://loschmidt.chemi.muni.cz
RESOURCES
Mendelian diseases dataset
A benchmark database for mendelian diseases

3. OUTPUT PAGE

Upon successful submission of a job, the user is redirected to the output page, where the following information is available:

1. **JOB INFORMATION** section – provides information whether the job is running or waiting in the queue. It also shows *Job title* and *Job ID* that can be used to access the job via search panel located in the header of the page. NOTE: Alternatively, you can either bookmark the whole page to access the job later, or provide an e-mail address on the input page.

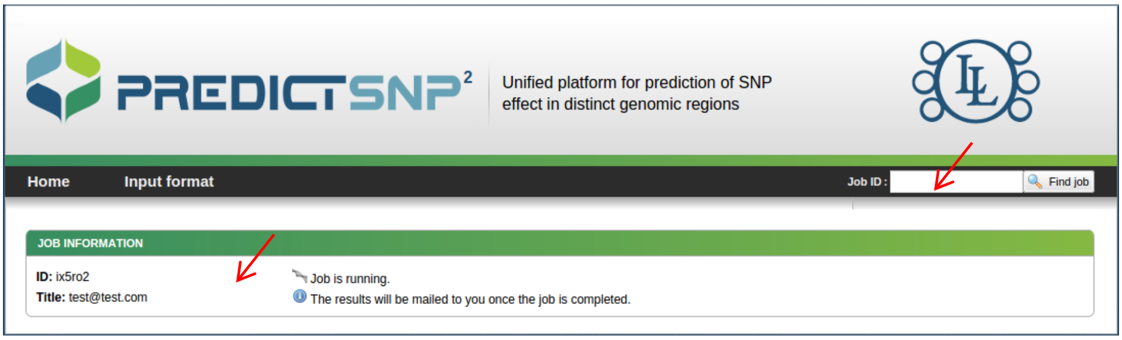

2. **RESULTS** section – once the job is finished, all results are shown in the summary table that is divided into three sub-sections: Input, Prediction tools and Databases. Input subsection contains the definitions of variants and corresponding regions and region functions detected by ANNOVAR. The predicted effect of PredictSNP consensus and individual tools is color-coded: neutral variants are in green, deleterious variants in red and undecided variants in gray. The normalized confidence of the tools is represented as a percentage corresponding to the observed accuracy measured for similar scores on the real data. The "-" symbol indicates that the respective variant was not properly evaluated by a given tool while "?" symbol indicates that the reported tool score has low confidence. When the mouse cursor is moved over any cell in the result table, a raw score of the corresponding tool is shown in a tooltip. The availability of database annotation for each variant is indicated by symbol. By clicking on the symbol, users are redirected to the original database record (mouse cursor over this symbol shows the description of the database). For any missense variants, the users can start the calculation of PredictSNP1 integrating six protein-based predictors by ticking the corresponding checkboxes in the last column of the table and clicking *Send to PredictSNP1* button below the table. Additionally, the users can switch between chromosome coordinates in *Genome assembly* box (GRCh37/hg19 and GRCh38/hg38 are currently supported).

   If the number of variants for analysis is larger than 50, only those predicted as the most deleterious according to the user-selected tool are shown in the result table (box *Prioritize by tool*; implicitly, PredictSNP2 tool is selected). The selection can be narrowed down to the specific category of variants, such as regulatory, splicing, missense, synonymous and nonsense subsets (box *Filter by category*).

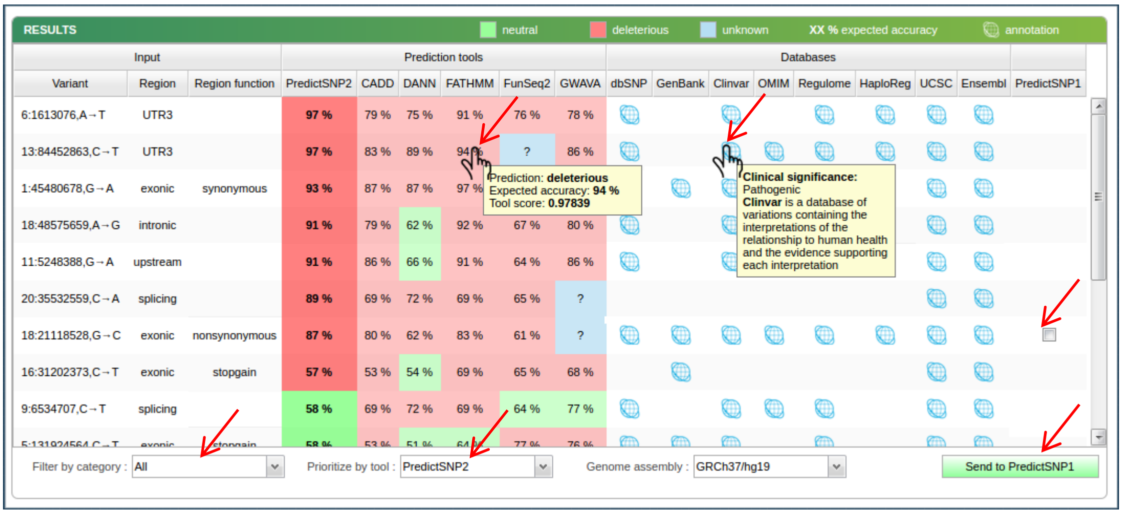

3. **DOWNLOAD** section – the results are available for download as VCF or PDF file. Note that a PDF file is generated for cases with less than 1,000 variants. In the case of problems, all errors are logged and accessible via *Error log* button.

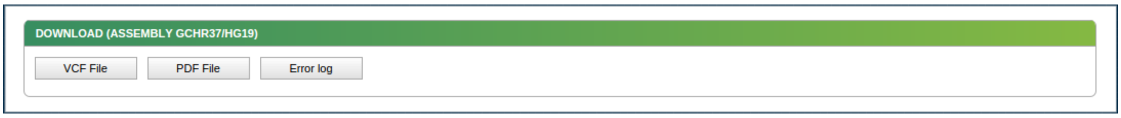

4. EXAMPLE

The PredictSNP2 server provides an embedded example, which can be raised by pressing *Load example* button in the INPUT section at the input page.

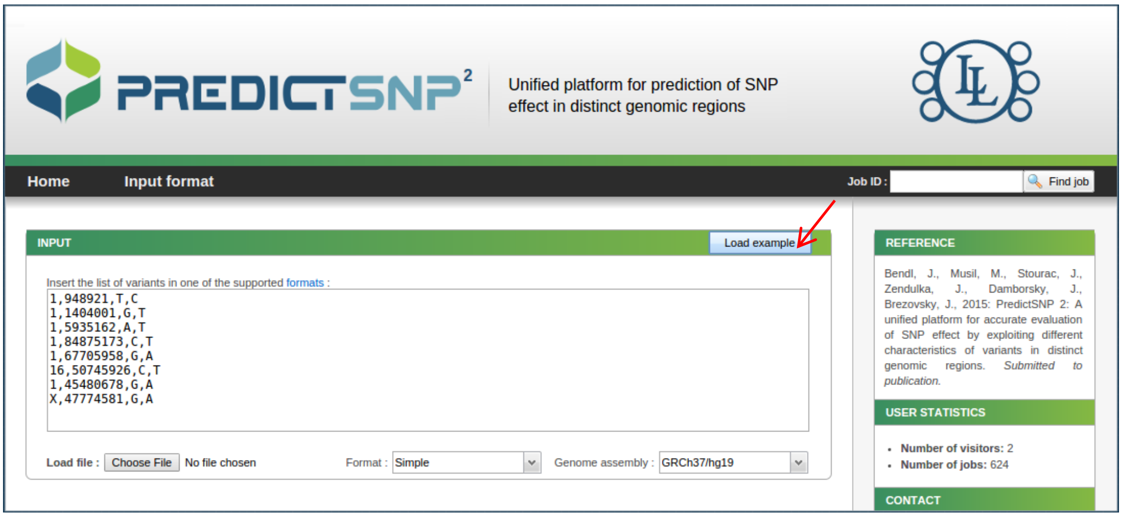

This loads the sequence of 18 variants located on 15 different chromosomes and 5 different regions (6 regulatory variants, 2 splicing variants, 3 missense variants, 4 synonymous variants and 3 nonsense variants). The experimental annotations from ClinVar database are available for 16 variants, out of which 9 variants are recognized as likely pathogenic / pathogenic and 7 variants as benign. The results of analysis provided by PredictSNP2 platform together with ClinVar annotations are summarized in the table on the next page. As can be seen from the table, the predictions of PredictSNP2 consensus score are also frequently in the correspondence with the experimental annotations. The variants, that are not annotated and at the same time are frequently predicted as deleterious with high confidence, might represent an interesting choice for an experimental study. Conversely, the mutations predominantly predicted as neutral might be deprioritized from a further study.

Chromosome	Position	rs# identifier	Reference allele	Observed allele	ANNOVAR annotation	ClinVar	Affected gene	Disease association	Prediction of tools [a]					
									PredictSNP2	CADD	DANN	FATHMM	FunSeq2	GWAVA
6	1613076	rs35717904	A	T	UTR3	Pathogenic	FOXC1	-	**97 %**	79 %	75 %	91 %	76 %	78 %
1	66075952	rs1805094	G	C	Exonic: nonsynonymous	Benign	LEPR	-	**89 %**	94 %	83 %	89 %	62 %	52 %
1	201328373	rs121964857	G	A	Exonic: nonsynonymous	Likely pathogenic	TNNT2	Cardiomyopathy	**65 %**	80 %	73 %	83 %	61 %	
18	48575659	rs377767327	A	G	Intronic	Pathogenic	SMAD4	Juvenile polyposis syndrome	**91 %**	79 %	62 %	92 %	67 %	80 %
3	37067050	rs11129748	A	G	Intronic	Benign	MLH1	-	**88 %**	86 %	82 %	93 %	68 %	64 %
11	5248388	rs33941377	G	A	Upstream	Pathogenic	HBB	Beta thalassemia	**91 %**	86 %	66 %	91 %	64 %	86 %
X	138642995	rs1800455	T	C	Exonic: synonymous	Benign	F9	-	**96 %**	95 %	97 %	73 %	93 %	54 %
18	21118528	rs80358257	G	C	Exonic: nonsynonymous	Pathogenic	NPC1	Niemann-Pick disease	**87 %**	80 %	62 %	83 %	61 %	
13	84452863	rs191284403	C	T	UTR3	Pathogenic	SLITRK1	Tourette syndrome	**97 %**	83 %	89 %	94 %		86 %
9	6534707	rs386833575	C	T	Splicing	Likely pathogenic	GLDC	Non-ketotic hyperglycinemia	**58 %**	69 %	72 %	69 %	64 %	77 %
20	35532559	not available	C	A	Splicing	-	-	-	**89 %**	69 %	72 %	69 %	65 %	
16	31202373	not available	C	T	Exonic: stopgain	-	-	-	**57 %**	53 %	54 %	69 %	65 %	68 %
4	70898922	rs17147990	T	A	Exonic: stopgain	Benign	HTN3	-	**81 %**	51 %	84 %	88 %	81 %	71 %
1	45480678	rs121918062	G	A	Exonic: synonymous	Pathogenic	UROD	Porphyria	**93 %**	87 %	87 %	97 %	93 %	54 %
10	124221276	rs2293870	G	T	Exonic: synonymous	Benign	HTRA1	-	**93 %**	83 %	87 %	80 %	93 %	60 %
5	131924564	rs373428259	C	T	Exonic: stopgain	Pathogenic	RAD50	Cancer-predisposing syndrome	**58 %**	53 %	51 %	64 %	77 %	76 %
2	47672475	rs1981928	T	A	Intronic	Benign	MSH2	-	**77 %**	76 %	54 %	85 %	68 %	74 %
17	7127146	rs35501596	G	A	Exonic: synonymous	Benign	ACADVL	-	**96 %**	79 %	97 %	72 %	93 %	68 %

[a] – predicted effect is color-coded: neutral variants are in green, deleterious variants in red, unknown / uncertain in gray
